# Disruption of ZC3H15 compromises telomere length maintenance by entrapping telomerase within cajal bodies

**DOI:** 10.1186/s13578-025-01449-z

**Published:** 2025-07-22

**Authors:** Chuanle Wang, Wei Chen, Ruofei Li, Yue Yang, Jiali Wu, Yuyang Tian, Zibin He, Song Lin, Xining Wang, Jianxi Zhu, Wenbin Ma, Zhou Songyang, Yan Huang

**Affiliations:** 1 MOE Key Laboratory of Gene Function and Regulation, State Key Laboratory of Biocontrol and Guangzhou Key Laboratory of Healthy Aging Research, School of Lifesciences, Sun Yat-Sen University, Guangzhou, 510275 China; 2 State Key Laboratory of Respiratory Disease, Guangzhou Medical University, Guangzhou, 511436 China; 3 Institute of Precision Medicine, The First Affiliated Hospital, Sun Yat-Sen University, Guangzhou, 510080 China; 4 Department of Nephrology, The First Affiliated Hospital, Sun Yat-Sen University, Guangzhou, 510080 China; 5 Department of Orthopaedics, The Seventh Affiliated Hospital of Sun Yat-Sen University, Shenzhen, 518107 China; 6 Hainan Academy of Medical Sciences, Hainan Medical University, Haikou, 571199 China

**Keywords:** ZC3H15, Telomerase, Cajal bodies, Cell senescence

## Abstract

**Background:**

Telomere homeostasis is pivotal in various biological processes including ontogeny, reproduction, physiological aging, and the onset of numerous diseases such as tumors. In human stem cells and approximately 85% of tumor cells, telomerase formed by TERT and TERC RNA complex is responsible for elongating telomeres. However, the intricate and precise regulatory mechanisms governing telomerase remain largely elusive.

**Methods and results:**

We developed a genome-wide trimolecular fluorescence complementation (TriFC) screen to identify TERC RNA-interacting proteins and found ZC3H15 (Zinc finger CCCH domain-containing protein 15) to interact with telomerase. ZC3H15 interacts with TERT via its N-terminal domain in an RNA-dependent manner. The proximity labeling technique PhastID revealed that ZC3H15 associates with proteins involved in regulation of ribonucleoprotein (RNP) complex biogenesis, snRNP assembly and RNA localization. Deletion of ZC3H15 upregulated telomerase activity but interestingly resulted in shortened telomeres and induced senescence in HTC75 cells, suggesting an unknown mechanism in regulating telomere length. Notably, we found ZC3H15 to associate with GEMs nuclear bodies, and its deletion led to the spatiotemporal fusion of GEMs and Cajal bodies, resulting in the sequestration of telomerase within Cajal bodies and a reduction in telomerase recruitment to telomeres during the S phase. Consistent with these findings, ZC3H15 ablation accumulated TERC precursor RNA.

**Conclusions:**

These observations provide valuable insights into the molecular mechanisms by which ZC3H15 regulates telomerase dynamics and cellular senescence. ZC3H15 may represent a new target for cancer treatment and anti-aging therapies.

**Supplementary Information:**

The online version contains supplementary material available at 10.1186/s13578-025-01449-z.

## Introduction

Telomeres are specialized structures located at the ends of linear chromosomes, composed of repetitive TTAGGG sequences and associated protein complexes known as shelterin or telosome [1, 2]. Their primary function is to protect chromosome ends from being recognized as double-strand breaks, thereby preserving genome stability [3]. During DNA replication, the semiconservative replication nature leads to the gradual shortening of telomeres, typically by approximately 150 base pairs per cell cycle [4, 5]. Eventually, when telomeres reach a critical length, known as the Hayflick limit, cellular proliferation halts [6]. The shortening of telomeres is closely associated with genome instability. The loss or dysfunction of telomeres can lead to chromosome fusion, breaks, and abnormal rearrangements, increasing genome instability, which is considered a significant driving factor in tumorigenesis [7, 8]. Studies have shown that genome instability not only promotes tumor formation but is also closely related to tumor progression and treatment resistance [7, 9, 10]. To counteract telomere shortening, approximately 85% of tumor cells employ telomerase for telomere elongation [11]. Telomerase is an RNP complex consisting of telomerase reverse transcriptase (TERT) protein and RNA component (TERC or TR) [3]. The interaction between the TEN domain of TERT and TPP1, a component of the shelterin complex, facilitates the recruitment of telomerase to telomeres [12, 13]. Subsequently, telomerase utilizes the RNA template within the complex to elongate telomeric DNA [3]. The maturation of stable TERC necessitates further processing at the 3′ end. Within the nucleus, the exosome complex was among the first regulatory entities identified to participate in TERC processing [14]. Subsequent research has elucidated that the exosome relies on several adaptor complexes, including NEXT, TRAMP, and PAXT, to recognize various RNA substrates [15, 16]. The NEXT complex consists of the scaffold protein ZCCHC8, the RNA helicase MTR4, and the RNA-binding protein RBM7 [16]. Previous studies have demonstrated that the knockout of ZCCHC8 or RBM7 results in TERC with 3′ extensions exceeding 50 nucleotides [14]. Moreover, the microRNA processor DGCR8 has been found to facilitate exosome-mediated degradation of TERC within the nucleolus [17]. Subsequent research on TERC regulation has increasingly focused on the antagonistic interaction between the TRAMP complex and the poly(A)-specific ribonuclease PARN, which represents a common maturation mechanism for many H/ACA snoRNAs [18]. The nucleolar-localized TRAMP complex, comprising MTR4, ZCCHC7, and the atypical poly(A) polymerase PAPD5 [16], facilitates the exosome-mediated degradation of TERC by adding poly(A) tails to its 3′ end [14, 19]. Due to the antagonistic interactions between PAPD5 and PARN, research has indicated that the knockdown of PAPD5 expression in cells from patients with PARN mutations can enhance the levels of mature TERC [20]. This partial restoration compensates for the telomerase activity deficit resulting from PARN mutations and mitigates the associated cellular phenotypes. These findings have catalyzed further investigations into the potential use of PAPD5 small molecule inhibitors as a therapeutic strategy for PARN mutation-related disorders, such as dyskeratosis congenita (DC) [21]. Our previous studies have demonstrated that cells deficient in the Cajal body-associated protein TOE1 exhibit a marked accumulation of TERC precursors bearing poly(A) tails and 3′ end extensions, which correlates with diminished telomerase activity and subsequent telomere shortening [22].

Nevertheless, the molecular mechanisms underlying the processing, assembly, and recruitment of telomerase remain elusive. In particular, the collaborative roles and division of labor between the GEMs bodies and the Cajal bodies in these processes remain largely unclear, although it is currently believed that telomerase maturation and assembly occur within Cajal bodies before being recruited to telomeres [23]. TERC RNA, serving as both a reverse transcription template and a scaffold molecule, plays a crucial role in the process of telomerase maturation and assembly [3]. Therefore, the screening of TERC-interacting proteins holds biological significance for a deeper understanding of telomerase maturation and assembly, as well as the molecular mechanisms regulating telomerase activity. Furthermore, TERC has been reported to modulate various biological functions beyond telomere maintenance. It is known to upregulate the expression of immune-related genes, activate the NF-κB signaling pathway, enhance cellular inflammation levels and immune response efficiency [24], and regulate other biological functions involved in tumor cell immune evasion [25]. Hence, the screening of TERC-interacting proteins aids in comprehending its broader biological functions. Currently, various techniques have been developed for screening of TERC-interacting proteins. One such technique is the MS2 system, which involves the insertion of the MS2 RNA aptamer into the RNA of interest, followed by targeting the MS2 coat protein (MCP) to these binding sites [26, 27]. This method allows for the specific isolation and study of particular RNA–protein complexes. Researchers have identified the RNA-binding protein HuR as a binding partner of TERC, which promotes the methylation of TERC at C106 and thereby facilitates the assembly of the TERC/TERT complex [28]. A method called RNA–protein interaction detection (RaPID) utilizes proximity biotinylation mediated by the BirA* biotin ligase [29]. This technique enables the rapid identification of proteins that bind to a specific RNA sequence of interest within living cells. By biotinylating proteins in close proximity to the RNA of interest, RaPID facilitates the subsequent isolation and identification of these RNA-binding proteins using streptavidin-based affinity purification, followed by mass spectrometry analysis [29]. This innovative approach offers a powerful tool for studying RNA–protein interactions in their native cellular context. Cas13 is a CRISPR-associated protein utilized for RNA targeting, and researchers have developed techniques for specific targeting and manipulation of RNA molecules using Cas13. Through precise engineering, Cas13 can be directed to target specific RNA sequences of interest, enabling detailed studies and potential therapeutic applications [30]. In general, these techniques provide powerful tools for in-depth exploration of interactions between telomerase and other proteins, offering new insights into the molecular mechanisms underlying telomere maintenance and related diseases.

Our laboratory pioneered a screening strategy based on bimolecular fluorescence complementation (BiFC) to systematically investigate protein–protein interactions within living cells [31]. This approach allowed for the comprehensive screening of telomerase regulatory factors. Trimolecular fluorescence complementation (TriFC) system, which is capable of screening for RNA-interacting proteins, was developed based on BiFC. Compared to traditional RNA pull-down assays combined with mass spectrometry identification, the TriFC technique can capture RNA–protein pairs with weaker and more transient interactions in live cells. Through high-throughput screening, we successfully identified several novel proteins that interact with TERC, offering new insights into the complex network of RNA–protein interactions. ZC3H15 was found to be a potential TERC interacting protein based on TriFC-based screening data. Found as a developmentally regulated GTP-binding protein, ZC3H15 is also named as DRG family regulatory protein 1 (DFRP1) [32]. Literatures have documented that ZC3H15 plays a pivotal role in facilitating the progression of multiple cancer types by safeguarding the stability of c-Myc and EGFR proteins [33, 34]. Due to few studies, the function of ZC3H15 is still not fully understood, especially its relationship with telomere homeostasis and aging. Here we elucidated the binding capability of ZC3H15 with telomerase. Deletion of ZC3H15 in HTC75 cells resulted in elevated telomerase activity, shortened telomere length, and increased levels of both overall and precursor TERC. Our newly developed proximity labeling technique PhastID [35] indicated that ZC3H15 is likely associated with translation and RNP complex biogenesis. The absence of ZC3H15 results in pronounced aberrant spatiotemporal fusion of GEMs and Cajal bodies, telomerase retention within Cajal bodies and subsequently diminishing its recruitment to telomeres during the S phase. In summary, our findings introduced a novel telomerase-interacting protein identified through high-throughput screening. We delineated its role in maintaining telomere homeostasis and restraining cellular aging, thereby offering a potential therapeutic target for cancer intervention and anti-aging research.

## Materials and methods

### Cell lines, plasmids, and siRNA

HEK293T, HeLa and HTC75 cells were cultured in Dulbecco's Modified Eagle Medium (DMEM) containing 10% (v/v) fetal bovine serum (FBS). The culture dishes were incubated in a 37 °C humidified incubator with 5% CO_2_. The full-length and truncated cDNAs of ZC3H15 and TERT were cloned into pDEST27 for GST tagging, pLenti for HA-FLAG (HAFL) tagging, and pET-28a for His-tagging. For knockdown experiments, shRNA sequences were inserted into the pLKO.1 lentiviral vector and then packaged into lentiviruses. Cell analyses were conducted 72 h post lentiviral infection. The shRNA sequences utilized in this study are as follows: shNC, 5′-CTTACGCTGAGTACTTCGA-3′; shZC3H15-1, 5′-CCTAGAATCAACAGGATGTTT-3′; shZC3H15-2, 5′-GAGCTGTTCAAACCTGTAGTT-3′; shZC3H15-3, 5′-GCTGACTTCAAAGCAGGGAAA-3′; siRNA oligos were ordered from RiboBio and transfected into cells using Lipofectamine® RNAi MAX (Thermo Fisher Scientific). siZC3H15-1: 5′-GAAGGAGAAGATTATCGAA-3′; siZC3H15-2: 5′-GGAGATAAGTGTAAGTTCT-3′. The RNAi negative control (siNC) was also purchased from RiboBio.

### T7E1 assay and generation of ZC3H15 KO cells mediated by CRISPR/Cas9

The T7 endonuclease I (T7E1) assay is a widely used method to detect genome editing events such as CRISPR/Cas9-induced mutations [36, 37]. Genomic DNA surrounding the target site is PCR amplified and denatured, allowing heteroduplex formation between wild-type and mutant DNA strands. T7E1, an endonuclease that recognizes and cleaves mismatched DNA, is then added to digest the heteroduplexes. Following digestion, the DNA fragments are separated and analyzed by gel electrophoresis to detect the presence of indels (insertions or deletions) resulting from non-homologous end joining (NHEJ) repair. The appearance of cleavage products indicates successful genome editing at the target locus. We designed three sgRNAs and established knockout ZC3H15 cell lines. The T7E1 digestion assay confirmed that the designed three sgRNA could effectively cleavage ZC3H15 genomic DNA. We conducted a transfection of two sgRNA-containing plasmids, sgRNA1-PX458 and sgRNA3-PX459, as well as sgRNA2-PX458 and sgRNA3-PX459, into HTC75 cells. Notably, PX458 harbors a GFP fluorescence signal, while PX459 harbors a DsRed signal. Through flow cytometry, we isolated monoclonal cells displaying both signals and expanded their cultures. Subsequently, western blotting was employed to assess the knockout efficiency. sg-ZC3H15-1: 5′-CACCGCGCAATGCCCCCCAAGAAAC-3′; sg-ZC3H15-2: 5′-CACCGAATGCACATACTACAGACTT-3′; sg-ZC3H15-3: 5′-CACCGTTTGGTAACATTTGGACCTA-3′.

### Analyzing TERC 3′ end using 3′ RACE

TERC 3′ end analysis was carried out utilizing the 3′ rapid amplification of cDNA ends (3′ RACE) method as described previously [38]. RT-PCR amplification was performed with the following primers: TERC-F: 5′-GGGAGGGGTGGTGGCCATTTTT-3′. TERC-R: 5′-CTACAACGATTAGAGTGCCTACAG-3′;

The 3′ RACE products were prepared for deep sequencing utilizing the TruSeq Nano DNA LT Library Prep kit (Illumina). The raw data comprised paired-end reads with a length of 250 bp. Adapter sequences were removed using Cutadapt (v1.14), and sequences of low complexity or quality were filtered out. The remaining reads were aligned to the hg19 TERC gene sequence and its surrounding 1 kb region using the MEM algorithm of bwa v0.7.10-r789 with default parameters. Pairwise alignment algorithms from the Biostrings package in R were employed to identify reads that perfectly matched TERC at the first base pair and the subsequent 18 bp, allowing ≤ 1 mismatch and no insertions/deletions. The count of left-end reads mapped to different positions at the 3′ end of TERC was determined, and their proportions relative to the total 3′ end counts of TERC were calculated. The raw data has been deposited in the SRA database under accession number SRR28975511.

### Immunofluorescence (IF) and combined immunofluorescence and fluorescence in situ hybridization (IF-FISH)

A 15 mm diameter cover glass was placed in a 12-well plate, and cells were inoculated onto it. After removing the medium, cells were washed with PBS three times, fixed with 4% paraformaldehyde (PFA) for 15 min, and washed again. Permeabilization was done with 0.5% Triton X-100 for 30 min, followed by three PBS washes. After blocking, cells were incubated with primary antibody diluent at 4 °C overnight in a wet box. Post-incubation, cells were washed with blocking solution, incubated with secondary antibody diluent in the dark for 45 min at room temperature, and finally stained with DAPI. For FISH procedures following immunofluorescence (IF-FISH), samples were dehydrated through an ethanol series (70%, 90%, 100%), heat-denatured, and hybridized with either a FITC-(CCCTAA)_3_ or Cy3-(TTAGGG)_3_ PNA probe (0.5 μg/mL). Nuclear DNA was counterstained with DAPI prior to imaging.

### qRT-PCR, q-TRAP, IP-TRAP and RNA immunoprecipitation (RIP)

Total RNA was extracted using TRIZOL and quantified with Nanodrop 1000. 1 μg of RNA was treated with RNase-free DNase I for 2 min at 42 °C, followed by reverse transcription using the RevertAid First Strand cDNA Synthesis kit with random hexamer or oligo(dT)_18_ primers. qPCR was performed on Applied Biosystems StepOne™ Real-Time PCR System using the ChamQ Universal SYBR qPCR Master Mix. Each 10 μl reaction contained 5 μl of qPCR Master Mix, 0.5 μl each of forward and reverse primers (10 μM each), 1 μl of cDNA, and 3 μl of H_2_O. The amplification protocol included an initial denaturation at 95 °C for 10 min, followed by 40 cycles of 95 °C for 15 s and 60 °C for 1 min. Quantification was conducted using the comparative CT (△△CT) method. The primer sequences utilized were: TERC FP: 5′-GGGAGGGGTGGTGGCCATTTTT-3′; TERC RP: 5′-GAACGGGCCAGCAGCTGACATT-3′; GAPDH FP: 5′-GGAGCGAGATCCCTCCAAAAT-3′; GAPDH RP: 5′-GGCTGTTGTCATACTTCTCATGG-3′.

The real-time quantitative PCR-based telomerase repeated amplification protocol (q-TRAP) analysis was executed in accordance with established methodologies [39]. Harvested cells were washed with PBS, followed by the addition of 200 μl NP-40 lysis buffer, and incubated on ice for 30 min. Post-incubation, the lysates were subjected to centrifugation at 14,000 rpm for 10 min at 4 °C. A 20 μl aliquot of the supernatant was extracted for protein concentration assessment. The q-TRAP reaction mixture was prepared as per the specified protocol [39]. Immunoprecipitation followed by telomerase repeated amplification Protocol assays (IP-TRAP) was performed following established procedures [40]. In summary, cell lysates were incubated with anti-FLAG agarose beads at 4 °C for 4 h. Subsequently, the eluates obtained from this immunoprecipitation step were utilized for q-TRAP analysis. RNA immunoprecipitation (RIP) assays were conducted following established protocols [41]. In brief, cell extracts were incubated with anti-FLAG agarose beads and subsequently washed four times with NETN buffer. RNA was then extracted using TRIZOL reagent. The isolated RNA was analyzed by real-time quantitative PCR utilizing ChamQ Universal SYBR qPCR Master Mix.

### Co-immunoprecipitation (co-IP), GST pull down and western blotting

Co-IP and GST pull down were performed using HEK293T cells transfected with two plasmids containing FLAG and GST tag respectively, typically within 36-48 h post-transfection. Cells were washed once with PBS and lysed on ice for 30 min with NETN lysis buffer. The lysates were centrifuged at 12,000 rpm for 10 min. A 20 μl aliquot of the supernatant was reserved as an input sample, while 200 μl of the supernatant was transferred to centrifuge tubes containing 20 μl of GST (17,075,601) or FLAG beads (L00432). The protein lysates were incubated with the beads at 4 °C for 2 to 4 h. Following immunoprecipitation, non-specific binding proteins were removed by washing the beads three times with NETN lysis buffer. Finally, 40 μl of NETN lysis buffer was added to the beads to prepare the pull down or IP samples. Western blotting was performed to analyze the interaction. Antibodies used in this study are: rabbit polyclonal anti-ZC3H15 (Proteintech, 26,241–1-AP), mouse monoclonal anti-FLAG (Abmart, M20008), mouse monoclonal anti-GAPDH (Proteintech, 60,004–1-Ig), rabbit polyclonal anti-GST (CST, 2622S), mouse monoclonal anti-TUBULIN (Sigma, T5168), mouse anti-LaminA/C (Abmart, SAB420023C). mouse anti-TERT (Santa Cruz, sc-7212). rabbit polyclonal anti-Coilin (Santa Cruz, H-300).

### RNA pull-down

RNA pull-down assay was performed following established procedures [42]. The experiment was conducted to assess the interaction between ZC3H15 and TERC. Following in vitro transcription, TERC was linearized and biotinylated. HEK293T cells were transfected with either GFP or ZC3H15, and the biotinylated TERC was incubated with cell lysates. Streptavidin magnetic beads were employed to capture the RNA–protein complexes, followed by thorough washing to remove unbound components. Western blotting was subsequently performed to detect ZC3H15 or GFP using a FLAG antibody, with GAPDH serving as a loading control.

### Terminal restriction fragment (TRF) assay

Cells in 6 cm dish were harvested and genomic DNA was extracted using the AxyPrep Blood Genomic DNA Small Volume Kit. Subsequently, enzymatic digestion was performed followed by electrophoresis on a 0.7% agarose gel at 5 V/cm for 12 h. The gel was then dried for 1 h. For alkali-denaturation, the gel was transferred to a hybridization tube and incubated twice with 10 mL of denaturation solution at 42 °C for 15 min each time. Following denaturation, the gel was neutralized twice with 10 mL of neutralization solution at 42 °C for 15 min each time. After neutralization, the gel was incubated with 10 mL of pre-hybridization solution at 42 °C for 30 min and hybridized overnight at 42 °C with 10 mL of hybridization solution containing the hybridization probe. Non-specific binding probes were washed away from the gel, and the gel was covered with a screen. After overnight incubation, the phosphorus screen was removed for scanning.

### Affinity capture of biotinylated proteins and identification by mass spectrometry

The procedure was executed as same as our prior studies [43]. In summary, 2–5 × 10^7^ cells were grown in 50 μM biotin for 16 h, then lysed using RIPA buffer. Lysates were incubated with streptavidin magnetic beads, subjected to reductive alkylation with DTT and IAA, digested with trypsin, and finally desalted using a C18 column. Mass spectra were acquired using the Orbitrap Fusion Lumos, and subsequent data analysis was performed utilizing MaxQuant_2.1.0.0 software [44]. The UniProt human 192,283 database was retrieved. Fraction of target (FOT) values, indicating the proportion of target signals in a sample, were calculated by dividing the iBAQ value [45] of the prey by the sum of all decoy iBAQ values, and were employed for data normalization.

### MS data screening and data analysis

Three independent samples were included in each group. Heatmaps were drawn using the R-pheatmap package (package version 1.0.12). To test the reliability of the data, Pearson correlation was calculated and visualized using R package corrplot (version 4.1.0). To do data filtering, only proteins that were identified by at least two unique peptides were retained. Then volcano plots were used to screen the enriched prey lists, with a criteria that a prey in the experimental group with an enrichment ratio over three times to that of the control group, and a p-value less than 0.05. Gene Ontology (GO) analysis of the enriched interactors were performed by Metascape (https://metascape.org/gp/index.html#/main/) [46], under default parameters and selected terms with q value (adjusted p-value using BH-adjustment) < 0.01. The terms were ranked by the order of fold enrichments and visualized by GraphPad prism 8. The proximity protein network was visualized by Cytoscape (version 3.8.2), using PPI information from STRING database (https://www.string-db.org/).

### Statistics

Experimental results, where applicable, were expressed as the mean ± standard deviation (SD) and are representative of at least three independent experiments. Statistical significance was evaluated using the Student's two-tailed unpaired t-test, with significance levels indicated as *P < 0.05, **P < 0.01, and ***P < 0.001.

## Results

### ZC3H15 is identified as a novel TERC RNA interacting protein through TriFC screening

**Fig. 1:**
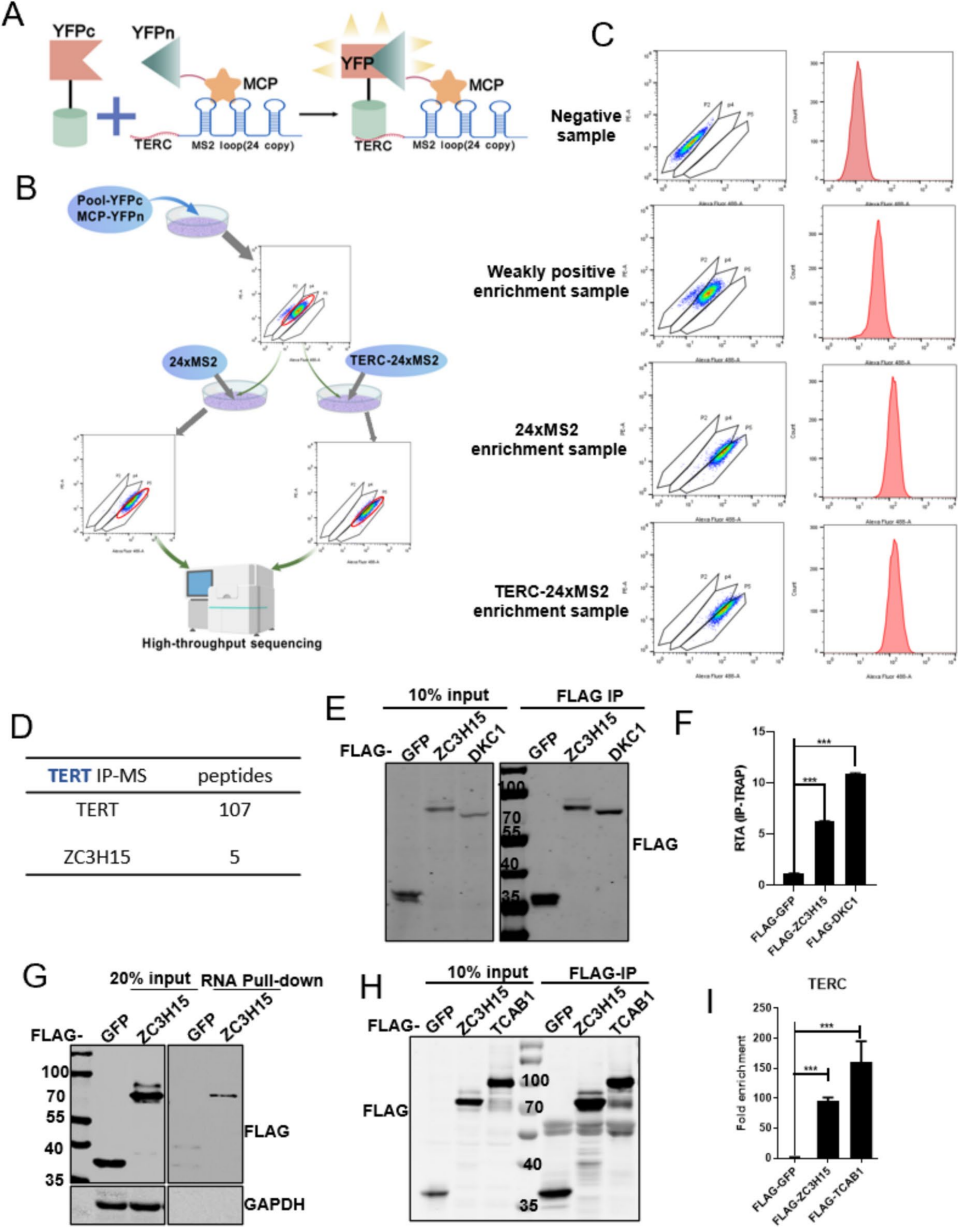
Identification of ZC3H15 as a novel TERC interacting protein through TriFC screening. **A** TriFC screening detects TERC-interacting proteins using split YFP. The N-terminal YFP is fused to the MS2 coat protein (MCP), which binds MS2 loops in TERC RNA. Interaction with a protein fused to the C-terminal YFP reconstitutes fluorescence, indicating a positive interaction. **B** The TriFC screening process pools cells expressing YFPc and MCP-YFPn constructs, then transfects with 24 × MS2 or TERC-24 × MS2. Flow cytometry detects fluorescence, indicating interactions. Positive interactions are sorted and analyzed by high-throughput sequencing to identify interacting proteins. **C** The flow sorting of TERC-interacting proteins in the TriFC system is shown with results from each sorting stage. The negative control is from the p2 region (TriFC platform screening cell library). The weakly positive sample is from the p4 region (after adding MCP). The strongly positive samples are from the p5 region (after adding 24 × MS2 or TERC-24 × MS2). Cells from the p5 region were expanded and collected for sequencing. **D** The TERT IP-MS data shows the number of peptide segments for the listed proteins. **E** and **F** ZC3H15 interacted with telomerase through IP-TRAP assay. IP efficiency was depicted (**E**) and telomerase activities were detected in anti-FLAG immunoprecipitated samples (**F**). RTA, relative telomerase activity. **G** Immunoblotting for specific correlation of ZC3H15 with TERC from RNA pull-down assays. **H** and **I** HEK293T cells transiently expressing FLAG-HA tagged GFP, ZC3H15 or TCAB1 were harvested for IP using anti-FLAG agarose beads. The IP samples were blotted with anti-FLAG antibodies (**H**). The relative TERC RNA level of IP from (**H**) was quantified by the RIP assay using qRT-PCR (**I**). Error bars represent S.D. (n = 3, technical replicates), *** P < 0.001, two-tailed unpaired t-test

The precise mechanisms of telomerase assembly and recruitment are not yet fully understood, though evidence suggests that these processes occur within Cajal bodies. Identifying proteins that interact with TERC is essential for elucidating the processes of telomerase maturation, assembly, and regulation. The principle of our TriFC screening is illustrated in Fig. 1A. We constructed YFP C-terminal fused to a cDNA library with 18,000 human open reading frames in HTC75 cell line stably transfected with YFP N-terminal fused MCP gene and obtained a weakly positive cell population through four rounds of enrichment (Fig. 1B, C). Subsequently, we stably transfected the TERC-24 × MS2 or a negative control 24 × MS2 gene into the weakly positive cell population and achieved a strongly positive YFP signal after seven rounds of enrichment (Fig. 1B, C). We then expanded the cell culture, extracted mRNA, and performed high-throughput sequencing. By analyzing the enriched gene list, we focused on ZC3H15 as our previous immunoprecipitation and mass spectrometry data of TERT detected ZC3H15 peptides (Fig. 1D). The specificity of the ZC3H15 antibody was assessed using western blot analysis in HEK293T cells overexpressed with SFB-tagged ZC3H15 vector and the empty control (Fig. S1A). Through nucleus/cytoplasm fractionation and immunofluorescence experiments, we found that although ZC3H15 is primarily localized in the cytoplasm, it is also in the nucleus (Fig. S1B, C). This nuclear localization supports its potential involvement in telomere regulation. We conducted immunoprecipitaion-telomere repeat amplification protocol (IP-TRAP) experiment and revealed the interaction between ZC3H15 and telomerase (Fig. 1E, F). Furthermore, RNA pull-down and RNA immunoprecipitation (RIP) experiments also demonstrated the interaction between ZC3H15 and TERC (Fig. 1G–I). These findings shed light on the potential involvement of ZC3H15 in telomerase function and telomere homeostasis, expanding our understanding of its cellular roles.

### ZC3H15 knockout results in increased telomerase activity and TERC levels

**Fig. 2:**
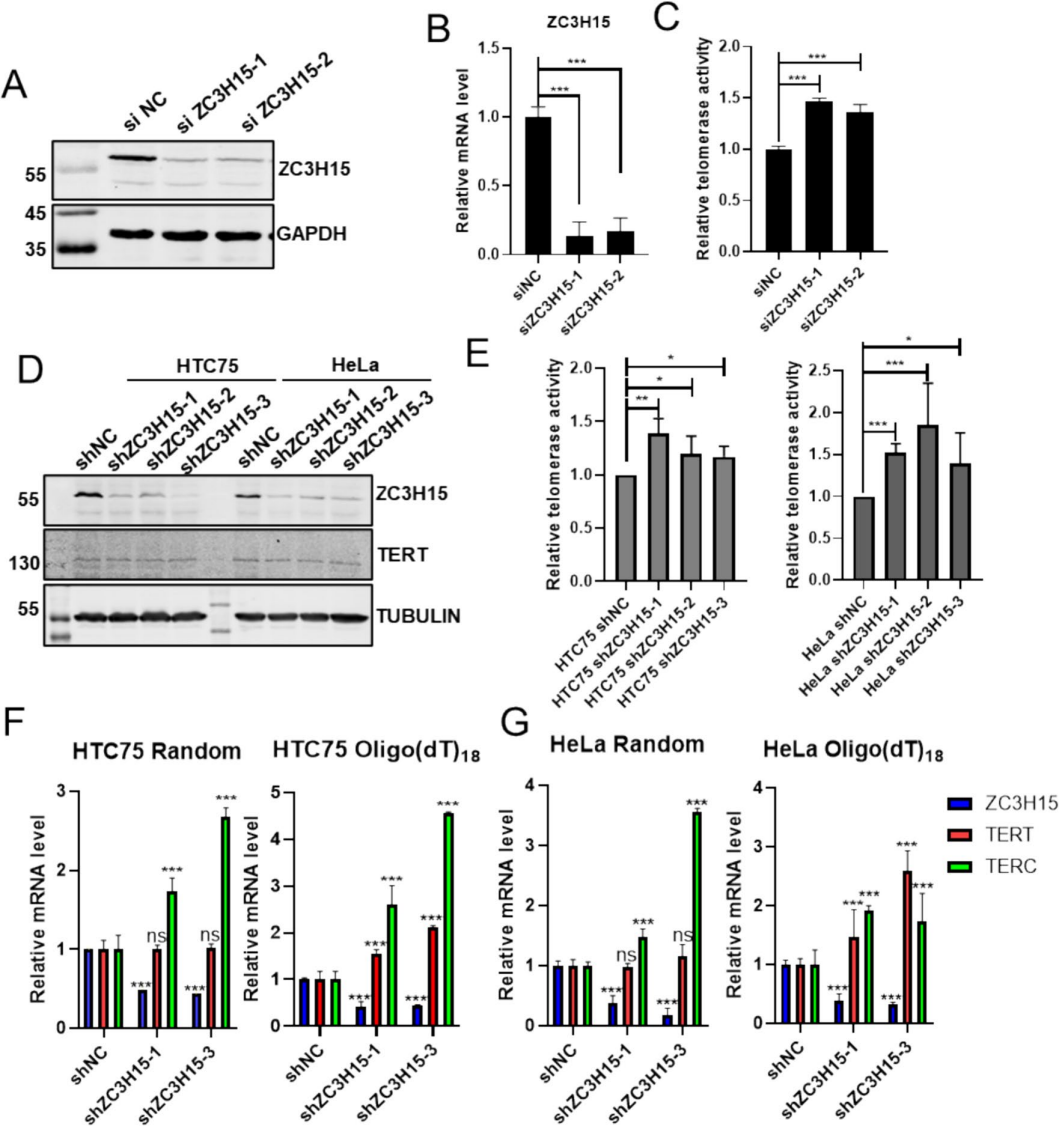
ZC3H15 depletion results in increased telomerase activity and TERC levels. **A** Two siRNAs targeting ZC3H15 were transiently transfected into HEK293T cells, with a non-targeting scramble siRNA serving as a negative control (NC). At 72 h post-transfection, knockdown efficiency was assessed by western blotting using antibodies against ZC3H15 and GAPDH. **B** RNA was extracted from cells from (**A**) for qRT-PCR. **C** The relative telomerase activity of cells from (**A**) was quantified by q-TRAP assay (n = 3, technical replicates). Error bars represent S.D., ***P < 0.001, two-tailed unpaired t-test. **D** Protein lysates of shRNA infected HTC75 and HeLa cells were subjected to immunoblotting using the specific antibodies indicated to detect the expression levels of the respective proteins. **E** The relative telomerase activity of cells from (**D**) was quantified by q-TRAP assay, error bars represent S.D. (n = 3, biological replicates). **F** and **G** RNA was extracted from shRNA infected HTC75 (**F**) and HeLa cells (**G**) for qRT-PCR analysis of TERC using hexamer or oligo (dT) primers. ns, no significance, ***P < 0.001, two-tailed unpaired t-test

To investigate the role of ZC3H15 on telomerase activity, we designed two specific siRNAs targeting ZC3H15. Western blot and qPCR results showed that both siRNAs significantly reduced ZC3H15 expression levels (Fig. 2A, B). Interestingly, knocking down ZC3H15 increased total telomerase activity in HEK293T cells (Fig. 2C). Stable ZC3H15 shRNA knockdown cell lines were also established in both HTC75 and HeLa cells (Fig. 2D). Consistent with the results obtained from transient transfection, ZC3H15 depletion led to an increase in telomerase activity (Fig. 2E). This corroborates ZC3H15’s involvement in the regulation of telomerase across different cellular contexts. We analyzed both the total TERC RNA and its precursors with the polyadenosine tail. Interestingly, we observed an overall upregulation in the expression of TERC RNA and its precursors upon ZC3H15 knockdown (Fig. 2F, G). Notably, although polyadenylated TERT mRNA levels seemed elevated under these conditions, neither reverse-transcribed TERT mRNA by random primer (Fig. 2F, G) nor TERT protein expression (Fig. 2D) exhibited significant changes. This indicates that ZC3H15 selectively influences the maturation or stability of TERC RNA without globally perturbing TERT protein expression.

### ZC3H15 knockout shortens telomere and induces cell senescence

**Fig. 3:**
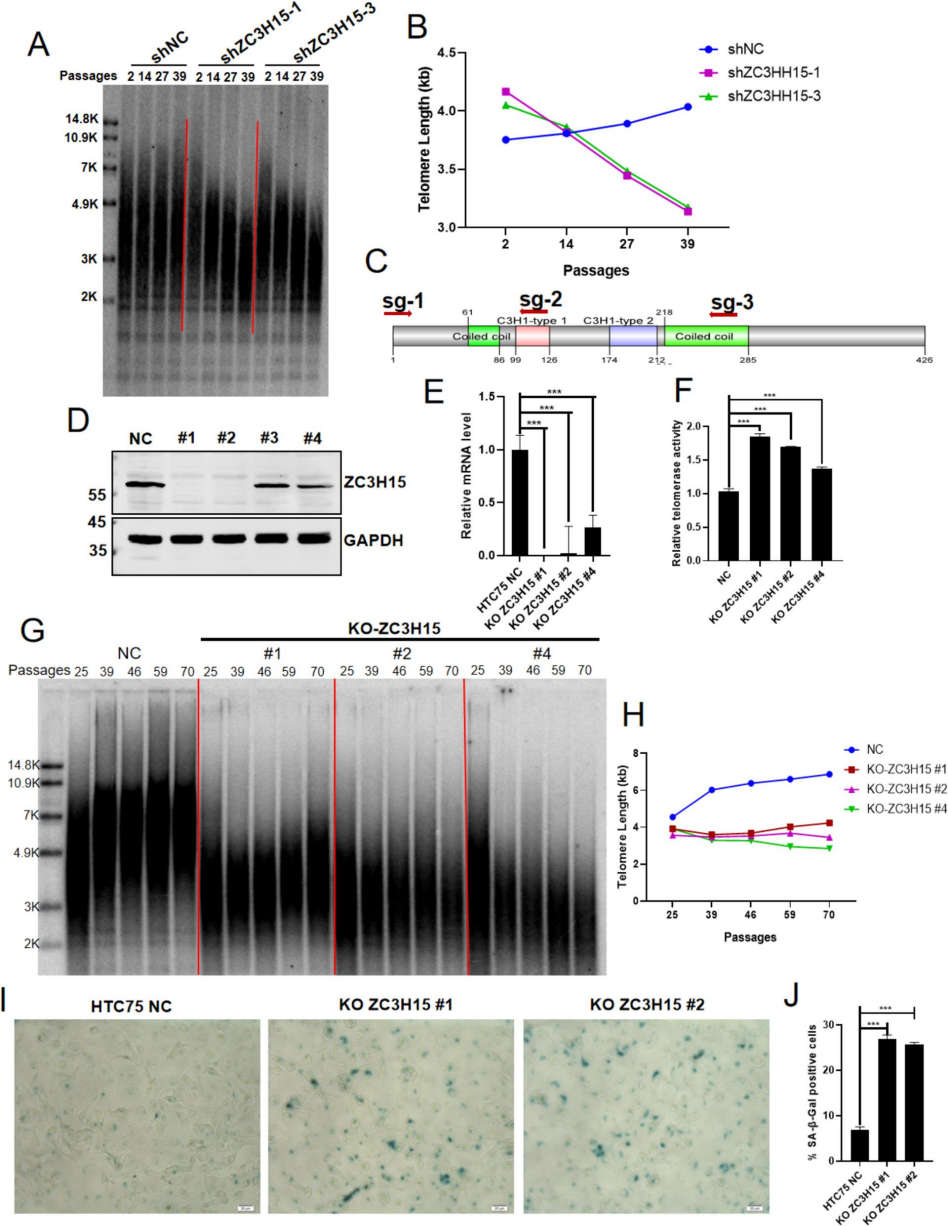
ZC3H15 depletion shortens telomere length and induces cell senescence. **A** In HTC75 cells, both the ZC3H15 knockdown and the negative control cells were passaged over time and harvested at the indicated time points for TRF analysis. PD, population doubling. **B** Quantification of data from (**A**) using ImageJ software. **C** Schematic representation of the dual gRNA targeting positions for knocking out ZC3H15. **D** The knockout (KO) efficiency of ZC3H15 in HTC75 cells was determined by western blotting using the indicated antibodies. **E** RNA was extracted from cells from (**D**) for qRT-PCR. **F** The relative telomerase activity of cells from (**D**) was quantified by q-TRAP. Error bars represent S.D. (n = 3, technical replicates). Significance was calculated using two-tailed unpaired t-test. ***P < 0.001. **G** Cells from (**D**) were passaged over time and harvested at the indicated time points for TRF analysis. PD, population doubling. **H** Quantification of data from (**G**) using ImageJ software. **I** and **J** Cells from (**D**) were stained for β-galactosidase activity (SA-β-gal), representative images were shown (**I**). The percentage of cells positive for SA-β-gal staining was quantified (**J**). For each group, at least 1200 cells were analyzed in a single repeated experiment. Error bars represented standard deviation (n = 3). P values were determined by Student’s t-test. ***, p < 0.001

We made the intriguing discovery that ZC3H15 knockdown results in higher telomerase activity. However, terminal restriction fragment (TRF) analysis yielded unexpected findings that ZC3H15 deletion correlates with telomere shortening in HTC75 cells (Fig. 3A, B). This unexpected observation suggests a nuanced role of ZC3H15 in telomere maintenance, warranting further investigation into its precise mechanisms and implications in cellular aging and disease progression. Due to the incomplete abolishment of ZC3H15 expression by shRNA, we utilized the CRISPR/Cas9 system to mediate the genetic knockout of ZC3H15. The T7E1 digestion assay confirmed that the designed three sgRNAs (Fig. 3C) could effectively cleave ZC3H15 genomic DNA (Fig. S1D). We conducted a dual sgRNA-mediated knockout strategy. Subsequently, western blotting (Fig. 3D) and qPCR (Fig. 3E) confirmed the knockout efficiency. Two monoclonal cell lines exhibiting complete knockout were successfully generated. Through q-TRAP analysis of telomerase activity, we observed that the deletion of ZC3H15 resulted in an upregulation of telomerase activity, consistent with that in ZC3H15 knockdown cells (Fig. 3F). Additionally, in line with previous findings, we also noted that ZC3H15 knockout led to increased levels of both total and precursor TERC transcripts (Fig. S1E). Genomic samples from ZC3H15 monoclonal cells were collected approximately every 10 generations during passages for telomere length analysis using TRF detection. In ZC3H15 knockout cells, telomere length persistently remained short across successive passages. Conversely, telomere length in the control cell line was longer and slightly extended with increasing passage numbers (Fig. 3G, H). This phenomenon suggested that the continuous absence of ZC3H15 during long-term monoclonal culture leads to telomere shortening. Once the telomeres reach a critical length, their shortening stabilizes (Fig. 3G, H). We also found that the deletion of ZC3H15 induces senescence in HTC75 cells (Fig. 3I, J). Previous studies reported that ZC3H15 interacts with TNF receptor-associated factor 2 (TRAF-2), a key signaling adaptor, and its depletion enhances MAPK activity and immune responses [47]. Consistent with these findings, our work demonstrates that ZC3H15 deficiency upregulates multiple immune-related genes and moderately elevates aging markers like senescence-associated secretory phenotype (SASP) factors (Fig. S2), suggesting its potential role in modulating cellular senescence through inflammatory regulation. In summary, our results indicated that both ZC3H15 knockdown and knockout result in telomere shortening.

### Accumulation of 3′-extended and oligoadenylated TERC in ZC3H15-deficient cells

**Fig. 4:**
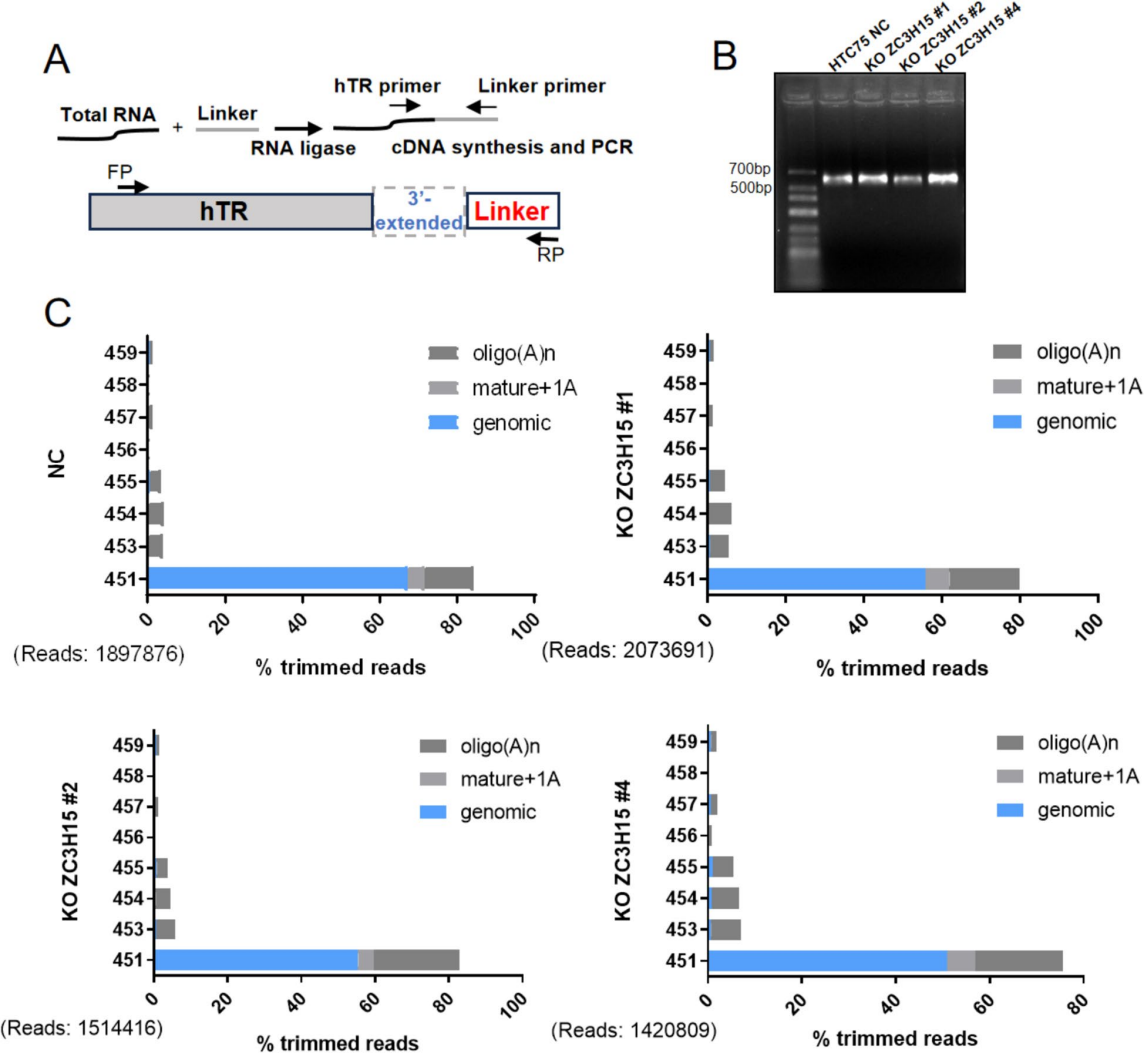
Accumulation of 3′-extended and oligoadenylated TERC in ZC3H15-deficient cells. **A** Strategy for TERC 3′ RACE. The primer set with F1 in the TERC and R1 in the linker was used to amplify TERC 3′RACE products. **B** TERC 3′ RACE PCR products from control (NC) and ZC3H15 KO HTC75 cells (KO1, KO2 and KO4) were resolved by agarose gel electrophoresis. **C** TERC 3′ RACE products from (**B**) were subjected to deep sequencing with reads aligned to the TERC gene. Genomically encoded termini are in blue, mature TERC with a single adenosine (which may be genomically encoded) is grey and oligo (**A**) additions (n ≥ 2) are in dark grey. The total number of trimmed reads for each group is shown in parentheses

Our investigation through qPCR analysis uncovered a significant elevation in the levels of oligo(A)-containing TERC in ZC3H15-deficient cells (Fig. 2F, G), implying a pivotal role of ZC3H15 in orchestrating the intricate process of 3′-end processing of TERC. To delve deeper into this phenomenon, we embarked on a comprehensive exploration utilizing 3′RACE and high-throughput sequencing techniques targeting the 3′-end of TERC specifically in ZC3H15 knockout cells (Fig. 4A). The ensuing RACE products revealed a striking increase in the proportion of immature TERC molecules bearing oligo(A) tails (Fig. 4B, C, S3), further corroborating our initial findings and suggesting a direct involvement of ZC3H15 in the regulation of TERC maturation at its 3′ end. These compelling results not only underscore the significance of ZC3H15 in telomere biology but also hint at its potential implications in cellular aging and disease progression. The intricate interplay between ZC3H15 and TERC processing mechanisms unraveled by our study provides valuable insights into the underlying molecular pathways governing telomere maintenance and cellular homeostasis.

### ZC3H15 interacts with TERT in an RNA-dependent manner

**Fig. 5:**
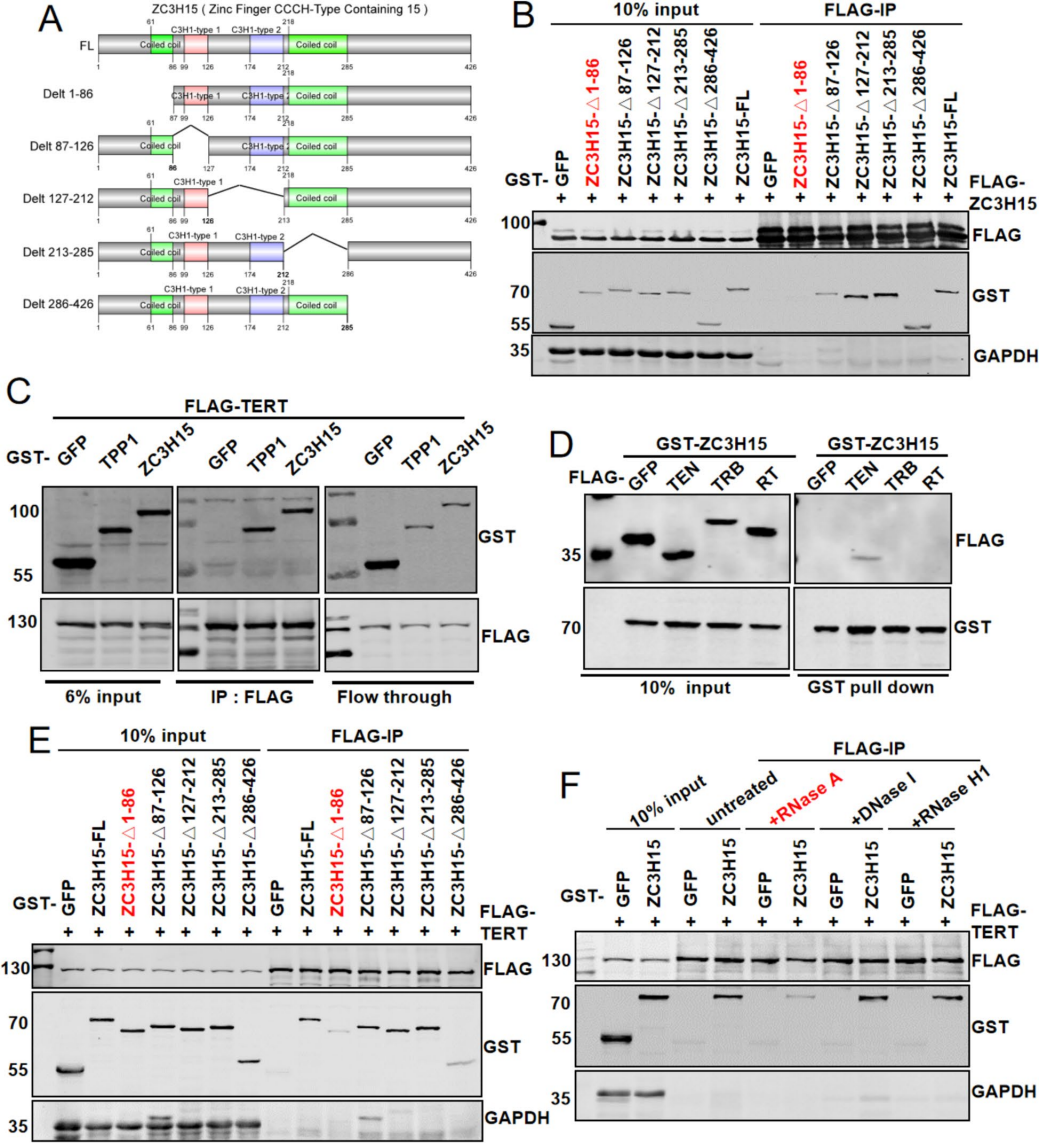
ZC3H15 interacts with TERT in an RNA-dependent manner. **A** Schematic diagram of full-length and truncated mutants of ZC3H15. **B** HEK293T cells transiently co-expressing HA-FLAG-tagged ZC3H15 along with GST-tagged ZC3H15 full length or truncated mutants were harvested for GST pull-down and immunoblotting with the indicated antibodies. **C** HEK293T cells transiently co-expressing FLAG-tagged TERT along with GST-tagged GFP, ZC3H15 or TPP1 were harvested for FLAG-IP and immunoblotting with the indicated antibodies. **D** HEK293T cells transiently co-expressing FLAG -tagged GFP or TERT truncation mutants along with GST-tagged ZC3H15 were harvested for GST pull-down and immunoblotting with the indicated antibodies. **E** HEK293T cells transiently co-expressing FLAG-tagged TERT along with GST-tagged ZC3H15 full length or truncated mutants were harvested for FLAG-IP and immunoblotting with the indicated antibodies. **F** HEK293T cells transiently co-expressing FLAG-tagged TERT and GST-tagged GFP or ZC3H15 were used for FLAG-IP in the presence or absence of RNase A

ZC3H15 exhibits self-interaction capacity (Fig. S4A). Structural analysis revealed an N-terminal coiled-coil domain (Fig. 5A), a conserved motif facilitating protein oligomerization. Consistent with this structural feature, the N-terminal domain mediates ZC3H15 self-association (Fig. 5B). Co-IP results showed that ZC3H15 interacts strongly with the telomerase protein TERT (Fig. 5C). To pinpoint the specific domain of ZC3H15 responsible for binding to TERT, we generated truncated mutants of ZC3H15 and TERT (unfortunately, the CTE domain of TERT failed to express). Our co-IP experiments revealed that the N-terminal TEN domain of TERT interacts with ZC3H15 (Fig. 5D). As mentioned in the foreword, TERT can bind to TPP1 through the TEN domain and then be recruited to telomeres. Therefore, we further explored whether ZC3H15 could affect the binding ability of TPP1 and telomerase, we transiently transfected FLAG-labeled TPP1 plasmid and GST-labeled GFP or ZC3H15 plasmids in HEK293T cells (Fig. S4B). After 48 h, the cells were collected for immunoprecipitation-telomerase activity analysis. As shown in Fig. S4C, overexpressed ZC3H15 did not affect the binding ability of TPP1 to telomerase. Additionally, we found that ZC3H15 primarily interacts with telomerase through its N-terminal domain (Fig. 5E). After RNase A treatment, the interaction between ZC3H15 and TERT was significantly weakened (Fig. 5F), indicating the crucial role of RNA molecules in stabilizing their binding.

### The interactome of ZC3H15 obtained by biotin proximity labeling technique PhastID

**Fig. 6:**
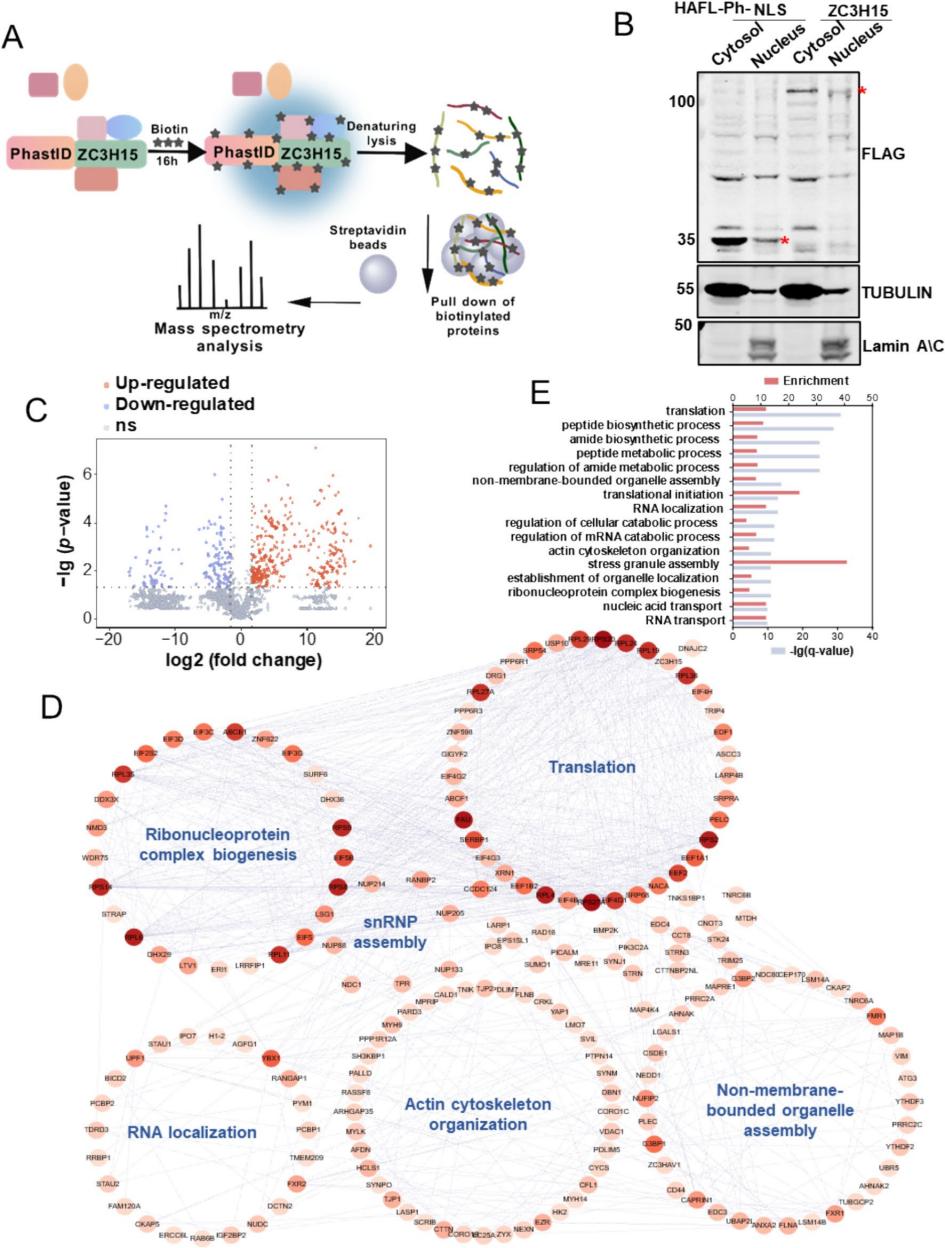
The interaction proteomics of ZC3H15 is obtained by biotin proximity labeling technique PhastID. **A** ZC3H15 was fused with the mutant biotin ligase PhastID and expressed in cells. Cells cultured with biotin would use the biotin ligase to biotinylate proteins in the vicinity of ZC3H15. Biotinylated proteins were then affinity purified and analyzed by liquid chromatography-tandem mass spectrometry (LC–MS/MS) to obtain the ZC3H15 proximity interaction network. **B** Nuclear extraction of PhastID-NLS and PhastID-ZC3H15 samples. The red stars indicate the sizes of the expected signals. **C** Volcano plot shows the identified proteins in the PhastID-ZC3H15 proximal interactors. Enriched proteins are defined as ≥ threefold change compared with PhastID-NLS with p-value ≤ 0.05 and highlighted in red, ns represents no significance. Blue denotes significantly downregulated proteins, gray represents non-significant hits. **D** Enriched ZC3H15-interacting protein network generated by Cytoscape, with node colors indicating the degree of interaction, where darker shades represent a higher number of interacting proteins. The network is organized into distinct functional clusters, including Translation, Ribonucleoprotein complex biogenesis, snRNP assembly, RNA localization, Actin cytoskeleton organization, and Non-membrane-bounded organelle assembly. Each node corresponds to a protein, and the connections between them illustrate their potential interactions within the ZC3H15 network. **E** Gene ontology analysis of proteins enriched by PhastID-ZC3H15. q value: adjusted p-value using BH-adjustment

To further explore the molecular mechanisms underlying ZC3H15’s regulation of telomerase activity and telomere length, we investigate the interactors of ZC3H15 by employing the biotin proximity labeling technique PhastID [35] coupled with mass spectrometry (Fig. 6A). Initially, we transfected PhastID-ZC3H15 (Ph-ZC3H15) constructs into HTC75 cells, cells expressing the biotin ligase PhastID fused with a nuclear localization signal (PhastID-NLS or Ph-NLS) were utilized as background controls (Fig. 6B). These cells presented expected biotin labeling signal upon biotin treatment (Fig. S5A). Biotinylated proteins were then affinity-purified and analyzed by mass spectrometry (Fig. 6A and S5B, C). Data were performed quality control and showed good reproducibility among three repeats (Fig. S5D, E). Proteins enriched by Ph-ZC3H15 were screened using volcano plot with ≥ threefold change, finally, 288 ZC3H15 inbred hetero-binding proteins in the nucleus were obtained (Fig. 6C). We observed core components of biological processes such as translation, RNP complex biogenesis, snRNP assembly, RNA location and non-membrane-bounded organelle assembly were enriched by Ph-ZC3H15 (Fig. 6D). This network underscores the intricate interconnectivity of ZC3H15 with diverse cellular functions. Gene ontology (GO) analysis presents a summary of the enrichment analysis results, delineating the biological processes associated with ZC3H15 interactions. Prominent processes include translation, peptide biosynthesis, and RNA transport, underscoring the multifaceted role of ZC3H15 in cellular metabolism and function (Fig. 6E). Collectively, these findings offer a comprehensive understanding of the functional landscape of ZC3H15, highlighting its pivotal involvement in numerous biological pathways.

### ZC3H15 knockout disrupts the spatiotemporal coordination of GEMs-Cajal bodies

**Fig. 7:**
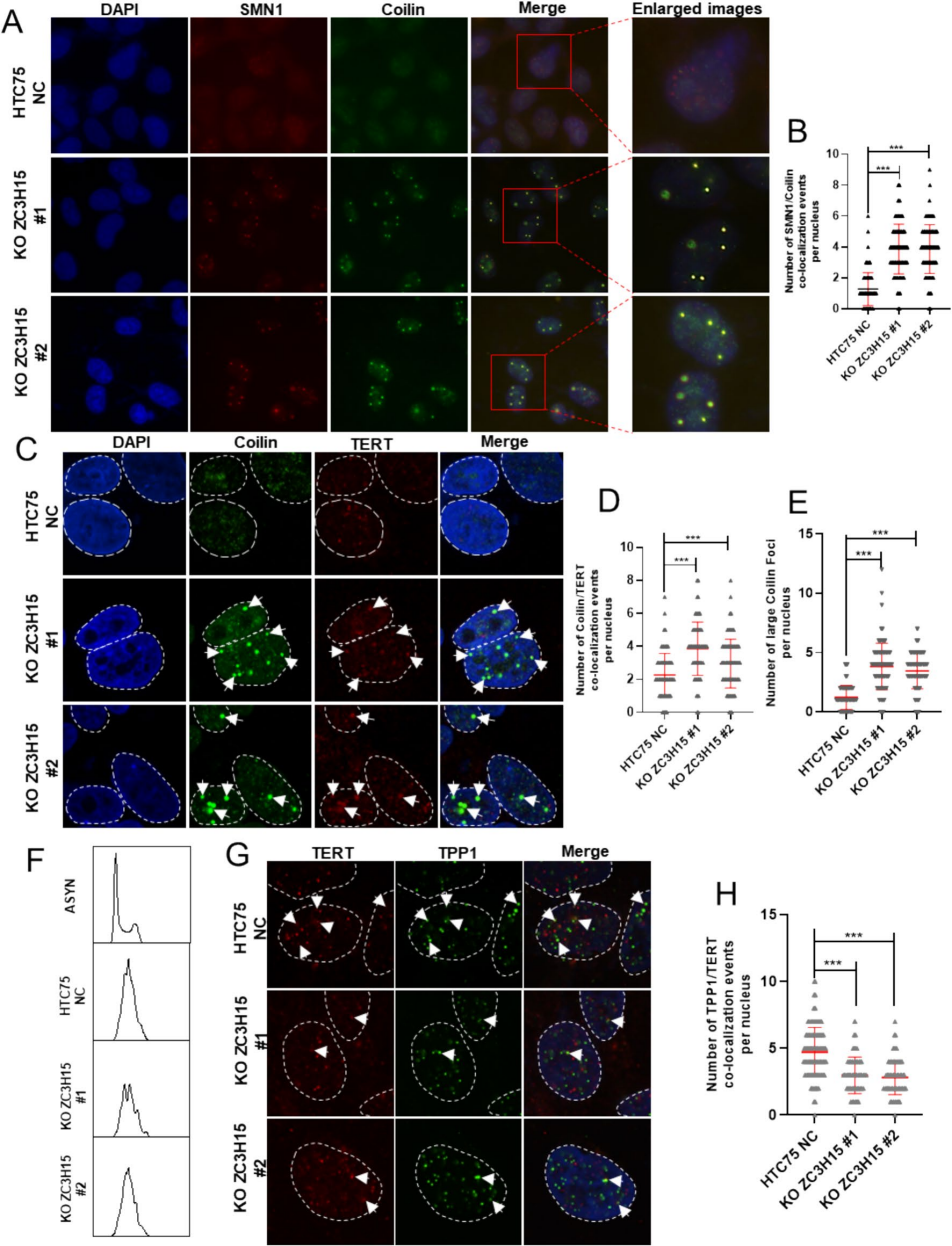
ZC3H15 knockout disrupts the spatiotemporal coordination of GEMs-Cajal bodies. **A** and **B** Negative control and ZC3H15 knockout HTC75 cells were stained with the Coilin antibody (green) and the SMN1 antibody (red). The enlarged images show characteristic co-localization signals (**A**). **B** Statistical representation of co-localization events of SMN1 and Coilin, quantified per nucleus. Approximately 200 cells were analyzed for each condition. Unpaired two-tailed Student’s t-test, *** P < 0.001. **C**–**E** Negative Control and ZC3H15 knockout HTC75 cells were stained with the Coilin antibody (green) and the TERT antibody (red) (**C**). The images shown illustrate the localization of Coilin in both the control (HTC75 NC) and ZC3H15 KO cells, with DAPI staining used to visualize the nuclei. White arrowheads indicate the co-stained signal. Fluorescence signal intensity was quantitated and the number of TERT foci co-localizing with Coilin signals per nucleus was plotted in **D**. The average number of large Coilin foci per nucleus in HTC75 cells was assessed in **E**. More than 120 cells were examined. Unpaired two-tailed Student’s t-test, *** P < 0.001. **F**–**H** Control and ZC3H15 knockout HTC75 cells were synchronized at the S phase of cell cycle by double-thymidine block. The cell cycle profile of the treated cells was analyzed by flow cytometry (**F**). Cells of each group underwent immunostaining with TPP1 antibody (green) and TERT antibody (red) (**G**). The colocalization of TPP1 and TERT was quantified (**H**). More than 150 cells were examined. Statistical analysis was performed using unpaired two-tailed Student’s t-test, ***P < 0.001

In light of the crucial roles that GEMs bodies and Cajal bodies play in the biological processes involving TERC, and given that proteomic interactions of ZC3H15 are linked to non-membrane-bounded organelle assembly (Fig. 6D, E), we sought to determine whether ZC3H15 is associated with these nuclear bodies. Immunofluorescence experiments revealed a higher degree of colocalization between ZC3H15 and SMN1, the core protein component of GEMs bodies (Fig. S5F, G). Consistent with these findings, co-IP results showed that ZC3H15 interact with SMN1 and Gemin7 (Fig. S5H, I), components of GEMs bodies, but does not show significant interaction with components of Cajal bodies (Fig. S5J). To investigate the impact of ZC3H15 on GEMs and Cajal bodies, we performed immunofluorescence and were surprised to find that the loss of ZC3H15 led to a strong fusion of GEMs and Cajal bodies (Fig. 7A, B). Since the GEMs and Cajal bodies are involved in the maturation of telomerase, we investigated the effect of ZC3H15 on telomerase assembly. Immunofluorescence analysis revealed an increased co-localization of telomerase with Cajal bodies in the ZC3H15-depleted cells, indicating telomerase retention in Cajal bodies (Fig. 7C, D). Additionally, the number and size of both GEMs bodies and Cajal bodies increased significantly (Fig. 7A, E). Nucleus/cytoplasm fractionation of ZC3H15-knockout and control HTC75 cells (Fig. S5K) revealed elevated nuclear TERC levels in ZC3H15-deficient cells by qRT-PCR analysis (Fig. S5L). This observation is particularly interesting because the coordinated involvement of GEMs and Cajal bodies in the biological process of telomerase assembly remains ambiguous. Concurrently, such a phenomenon raises intriguing questions about the potential role of ZC3H15 in modulating TERC processing and telomerase activity within GEMs and Cajal bodies, possibly implicating a regulatory mechanism in telomerase function. Given the observed obstruction of telomerase within Cajal bodies, our investigation delved further into the dynamics of telomerase recruitment. To elucidate this, we initiated a double block of TDR to synchronize cellular progression into the S phase (Fig. 7F). Remarkably, a significant reduction in the recruitment of telomerase in the S phase to telomeres was observed upon ZC3H15 knockout (Fig. 7G, H and S5M, N). Given the critical role of shelterin proteins in the recruitment of telomerase, we sought to determine whether ZC3H15 collaborates with these proteins in this process. Our findings indicate that ZC3H15 does not interact with shelterin proteins (Fig. S5O). This observation underscores the pivotal role of ZC3H15 in orchestrating the intricate process of TERC processing and telomerase recruitment, further emphasizing its significance in telomere maintenance and cellular homeostasis.

These findings highlight the multifaceted role of ZC3H15 in cellular processes, potentially extending beyond its previously known functions. Further elucidating the molecular mechanisms underlying ZC3H15’s interactions with telomerase and TERC maturation could provide valuable insights into cellular homeostasis and potentially uncover novel therapeutic targets for diseases associated with telomere dysfunction.

## Discussion

TERC, or telomerase RNA component, plays a crucial role in cellular biology, particularly in maintaining telomere length and stability. TERC provides the template for TERT to synthesize telomeric DNA repeats, counteracting the progressive telomere shortening that occurs with each round of cell division, thus preserving chromosomal integrity and cellular viability [3]. Understanding the biological functions of TERC is crucial for unraveling the mechanisms behind telomere maintenance and their implications in aging, cancer, and other age-related diseases. Therefore, identifying proteins that interact with TERC is of significant importance in elucidating the regulatory networks governing telomerase activity and telomere biology. Our study elucidates the critical role of ZC3H15 in telomerase maturation, assembly and recruitment. Using the TriFC genome-wide screen system, we identified the interaction between ZC3H15 and telomerase (Fig. 1D–F). The deletion of ZC3H15 resulted in increased telomerase activity (Fig. 3F) but caused telomere shortening (Fig. 3A, G) and induced cellular senescence (Fig. 3I, J) in HTC75 cells. Additionally, the absence of ZC3H15 led to elevated expression levels of TERC and its precursor RNA (Fig. 2F, G). More importantly, we found that the knockout of ZC3H15 induces large-scale fusion of GEMs and Cajal bodies (Fig. 7A, B) and diminishes telomerase recruitment to telomeres (Fig. 7G, H). Consequently, this disruption precipitates telomere shortening and cellular aging.

The subcellular localization of telomerase assembly remains a topic of debate. It is commonly posited that telomerase assembly occurs in the nucleolus and Cajal bodies, given that TERC is enriched in these regions [23]. WRAP53 can specifically bind to the CAB box of TERC, recruit TERC to Cajal bodies, and facilitate the assembly and trafficking of telomerase [23]. Consequently, Cajal bodies are proposed to be the primary sites for telomerase assembly. However, studies have demonstrated that knocking out the Cajal body scaffold protein Coilin does not impact the interaction of telomerase components, telomerase activity, telomere recruitment, or telomere length [48]. This observation implies the presence of alternative mechanisms, independent of Cajal bodies, that assist in the assembly of the telomerase catalytic subunit TERT and the template component TERC. The Gem localized SMN1 protein is known to be associated with telomerase. Using in vitro GST pull-down and co-IP assays, an association between SMN1 and the telomerase RNP was established, indicating that SMN1 interacts with a subset of the functional telomerase holoenzyme [49]. Overexpression of the SMN1 protein mutant, SMN∆27, disrupts the nuclear localization of TERT, resulting in increased cytoplasmic accumulation of TERT and a concomitant reduction in telomerase reconstitution activity in vitro [50]. Furthermore, studies have demonstrated that the SMN1 protein can interact with the telomerase TERC-binding protein GAR1. This interaction is dependent on the Tudor domain of SMN1 and the two RG-rich GAR domains of GAR1, although the arginine methylation status of the GAR domains does not influence their binding [51, 52].

Previous research has established that GEMs and Cajal bodies are involved in the biogenesis and assembly of telomerase [23, 49]. Our study adds to this knowledge by identifying ZC3H15 as a key regulator in this process. Most GEMs and Cajal bodies exist as independent nuclear bodies in fetal tissues, but their colocalization becomes increasingly evident with fetal age. In adult cells, GEMs and Cajal bodies often lie close to each other or even completely overlap [53]. In most human cell lines, the foci of Coilin and SMN1 partially overlap or completely coincide, leading some researchers to speculate that GEMs and Cajal bodies may represent two parts of the same nuclear structure [54]. However, other studies suggest that GEMs are a distinct type of nuclear body [55, 56]. Unlike prior studies that did not establish a direct link between ZC3H15 and these nuclear bodies, our findings highlight the essential role of ZC3H15 in maintaining the structural and functional integrity of GEMs and Cajal bodies. ZC3H15 deletion leads to the sequestration of telomerase within Cajal bodies (Fig. 7C, D) and a reduction in its recruitment to telomeres during the S phase (Fig. 7F–H). Nonetheless, the precise molecular mechanisms by which ZC3H15 regulates TERC processing and telomerase assembly require further investigation.

Previous studies have reported that knockout of ZC3H15 activates immune pathways [47]. Our research suggests that cells may sequester aberrant RNA into newly formed nuclear bodies, thereby preventing the downstream activation of the type I interferon signaling pathway. This association suggests diverse functions of ZC3H15 within cells, potentially impacting immune responses through regulation of RNA metabolism and transport. Our study demonstrates that ZC3H15 knockout results in elevated levels of precursor RNA and telomerase aggregation into Cajal bodies. This phenomenon suggests that the increased number of Cajal bodies may serve as temporary storage sites for aberrant RNA, thereby preventing their activation of immune pathways within cells. ZC3H15 may regulate the TERC processing and telomerase assembly through modulating the formation and communication of GEMs and Cajal bodies. Future research should focus on elucidating the detailed molecular mechanisms by which ZC3H15 regulates telomerase activity and the biogenesis of GEMs and Cajal bodies. Additionally, further investigation is warranted to understand how ZC3H15 mediates inter-nuclear body communication and cellular stress responses to prevent immune response-induced cell death or senescence. Examining the role of ZC3H15 in various cell types and in vivo models will help confirm its broader biological significance. Moreover, exploring the potential therapeutic strategy targeting ZC3H15 for treating diseases related to telomerase dysfunction, such as cancer and aging-related disorders, is imperative. Consistantly to our results, lower level of ZC3H15 correlates with a better survival outcome of cancer patients (Fig S6).

## Conclusions

In conclusion, our study reveals that ZC3H15 plays a critical role in regulating telomerase maturation and telomere dynamics. ZC3H15 interacts with telomerase in an RNA-dependent manner, and its deletion leads to dysregulated telomerase activity, shortened telomeres, and premature cellular senescence. Most strikingly, ZC3H15 depletion triggers coalescence of two distinct nuclear bodies (GEMs and Cajal bodies), constituting our most pivotal discovery. Additionally, ZC3H15 is involved in the proper localization and assembly of telomerase in Cajal bodies, with its absence impairing telomerase recruitment to telomeres during the S phase. These findings highlight ZC3H15 as an important regulator of telomere homeostasis, with potential implications for cancer therapy and anti-aging interventions.

## Supplementary Information

Additional file 1.

## Data Availability

All raw data are available upon request. The mass spectrometry proteomics data have been deposited to the ProteomeXchange Consortium (https://proteomecentral.proteomexchange.org) via the iProX partner repository [57, 58] with the dataset identifier PXD052036. The 3′ RACE raw data has been submitted to the SRA database under accession number SRR28975511.

## Abbreviations

BiFC: Bimolecular fluorescence complementation
Co-IP: Co-immunoprecipitation
DFRP1: DRG family regulatory protein 1
DMEM: Dulbecco’s Modified Eagle Medium
GO: Gene Ontology
IF: Immunofluorescence
NLS: Nuclear localization signal
PhastID: *Pyrococcus* *horikoshii* BPL-assisted identification
q-TRAP: Real-time quantitative PCR-based telomerase repeated amplification protocol
RACE: Rapid amplification of cDNA ends
RaPID: RNA–protein interaction detection
RIP: RNA co-immunoprecipitation
RNP: Ribonucleoprotein
TERC: Telomerase RNA component
TERT: Telomerase reverse transcriptase
TRF: Terminal restriction fragment
TriFC: Trimolecular fluorescence complementation
ZC3H15: Zinc finger CCCH domain-containing protein 15
