# Supplementary material for: Disruption of ZC3H15 compromises telomere length maintenance by entrapping telomerase within cajal bodies
Source: Cell Biosci. 2025 Jul 22;15:107. doi: 10.1186/s13578-025-01449-z (PMC12285200; doi:10.1186/s13578-025-01449-z)
Supplement: Supplementary file 1 — Additional file 1. [file 13578_2025_1449_MOESM1_ESM.pptx]

## Slide 1
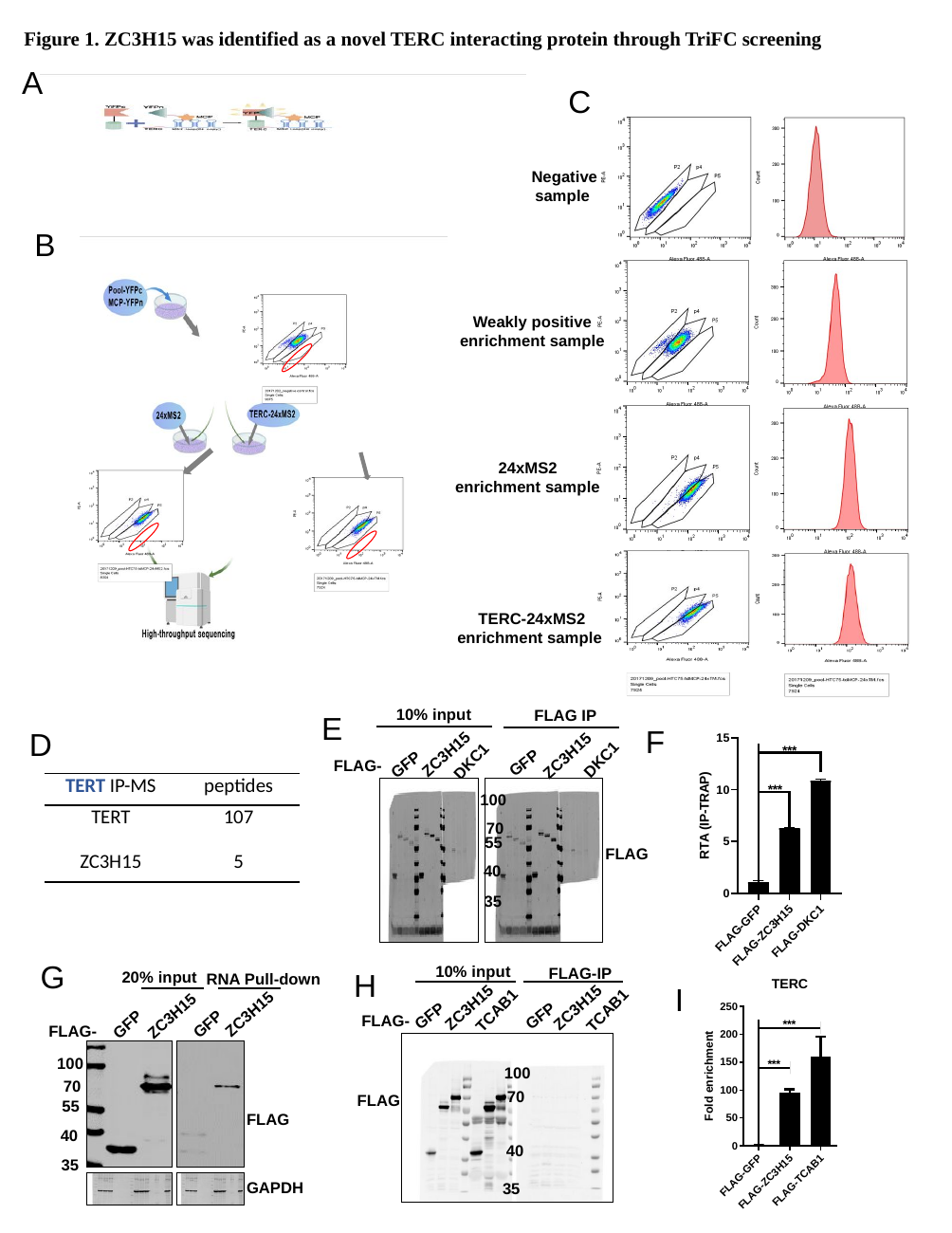

Figure 1. ZC3H15 was identified as a novel TERC interacting protein through TriFC screening
A
C
Negative sample
Weakly positive
enrichment sample
24xMS2
 enrichment sample
TERC-24xMS2 enrichment sample
B
F
10% input
FLAG IP
E
F
D
ZC3H15
ZC3H15
DKC1
DKC1
GFP
GFP
FLAG-
| TERT IP-MS | peptides |
| --- | --- |
| TERT | 107 |
| ZC3H15 | 5 |
100
70
55
40
35
FLAG
10% input
FLAG-IP
ZC3H15
ZC3H15
TCAB1
TCAB1
GFP
GFP
FLAG-
100
70
40
35
FLAG
G
20% input
RNA Pull-down
ZC3H15
ZC3H15
GFP
GFP
FLAG-
FLAG
GAPDH
H
I
100
70
55
40
35

## Slide 2
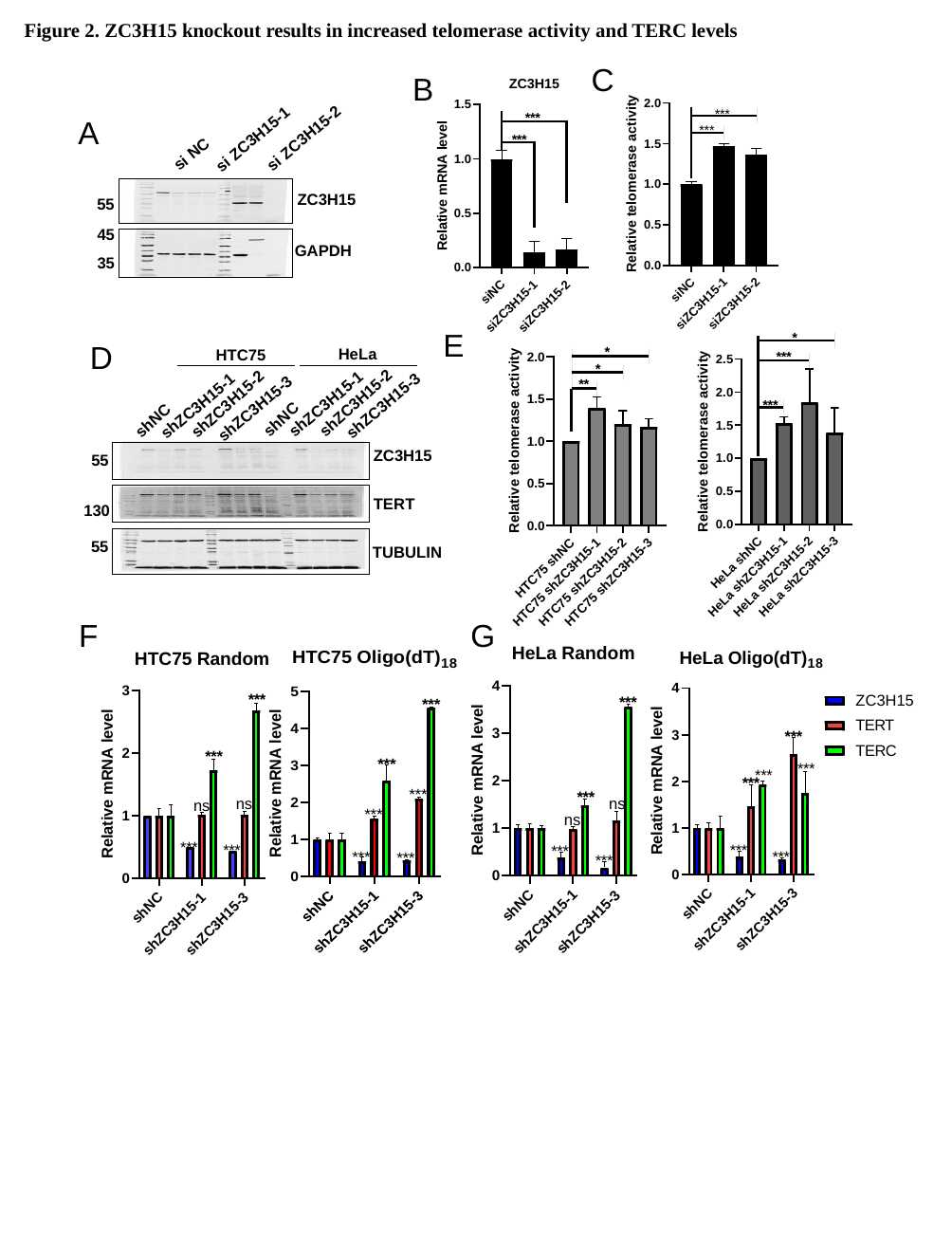

Figure 2. ZC3H15 knockout results in increased telomerase activity and TERC levels
C
B
A
si ZC3H15-2
si ZC3H15-1
si NC
ZC3H15
55
45
GAPDH
35
E
HeLa
HTC75
shZC3H15-2
shZC3H15-1
shZC3H15-2
shZC3H15-3
shZC3H15-1
shZC3H15-3
shNC
shNC
ZC3H15
TERT
TUBULIN
D
55
130
55
F
G

## Slide 3
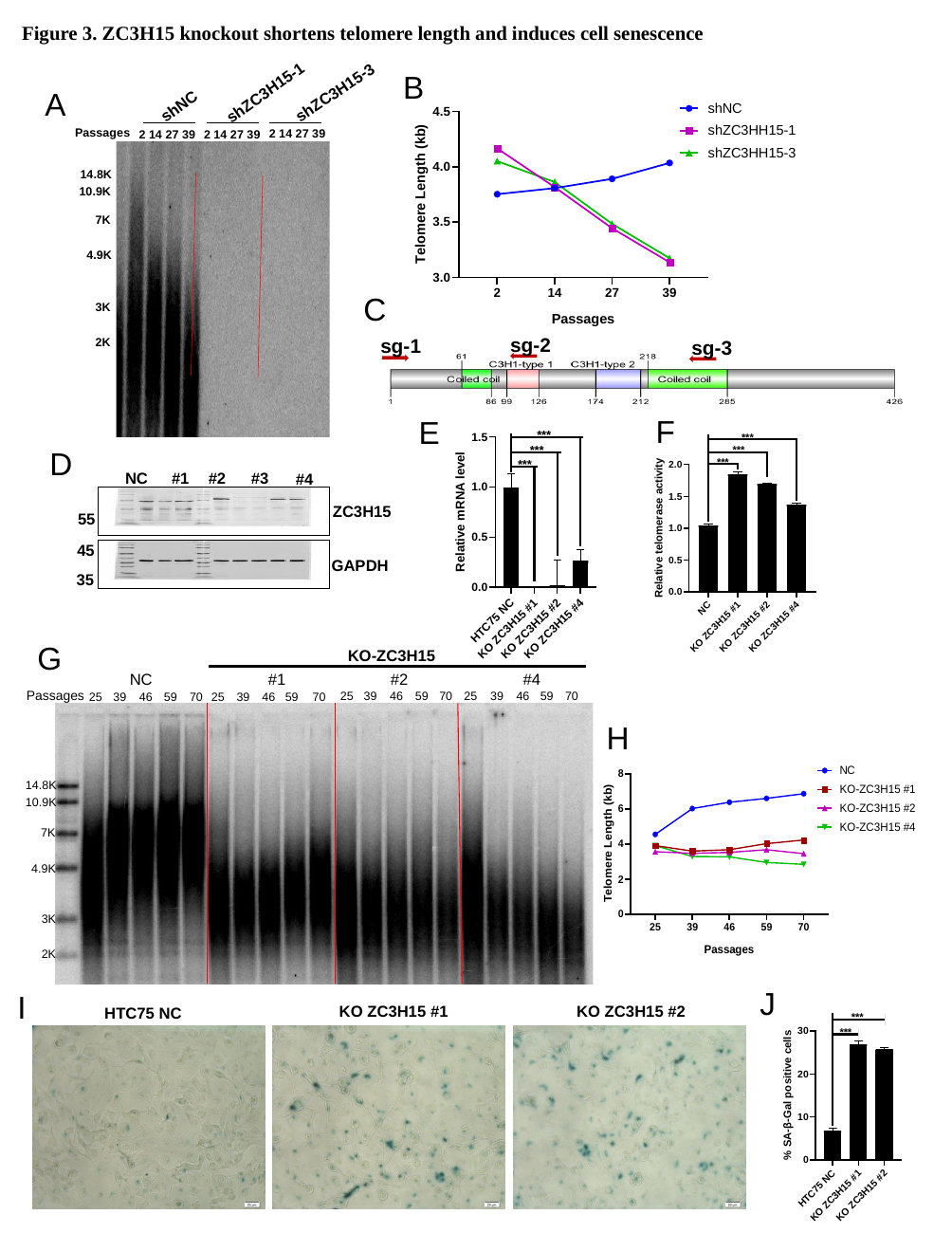

Figure 3. ZC3H15 knockout shortens telomere length and induces cell senescence
B
shZC3H15-1
shZC3H15-3
A
shNC
Passages
2 14 27 39
2 14 27 39
2 14 27 39
14.8K
10.9K
7K
4.9K
3K
2K
C
sg-2
sg-1
sg-3
F
E
D
NC
#2
#3
#1
#4
ZC3H15
GAPDH
55
45
35
G
KO-ZC3H15
NC
#1
#2
#4
Passages
25
39
46
59
70
25
39
46
59
70
25
39
46
59
70
25
39
46
59
70
14.8K
10.9K
7K
4.9K
3K
2K
H
J
I
KO ZC3H15 #2
KO ZC3H15 #1
HTC75 NC

## Slide 4
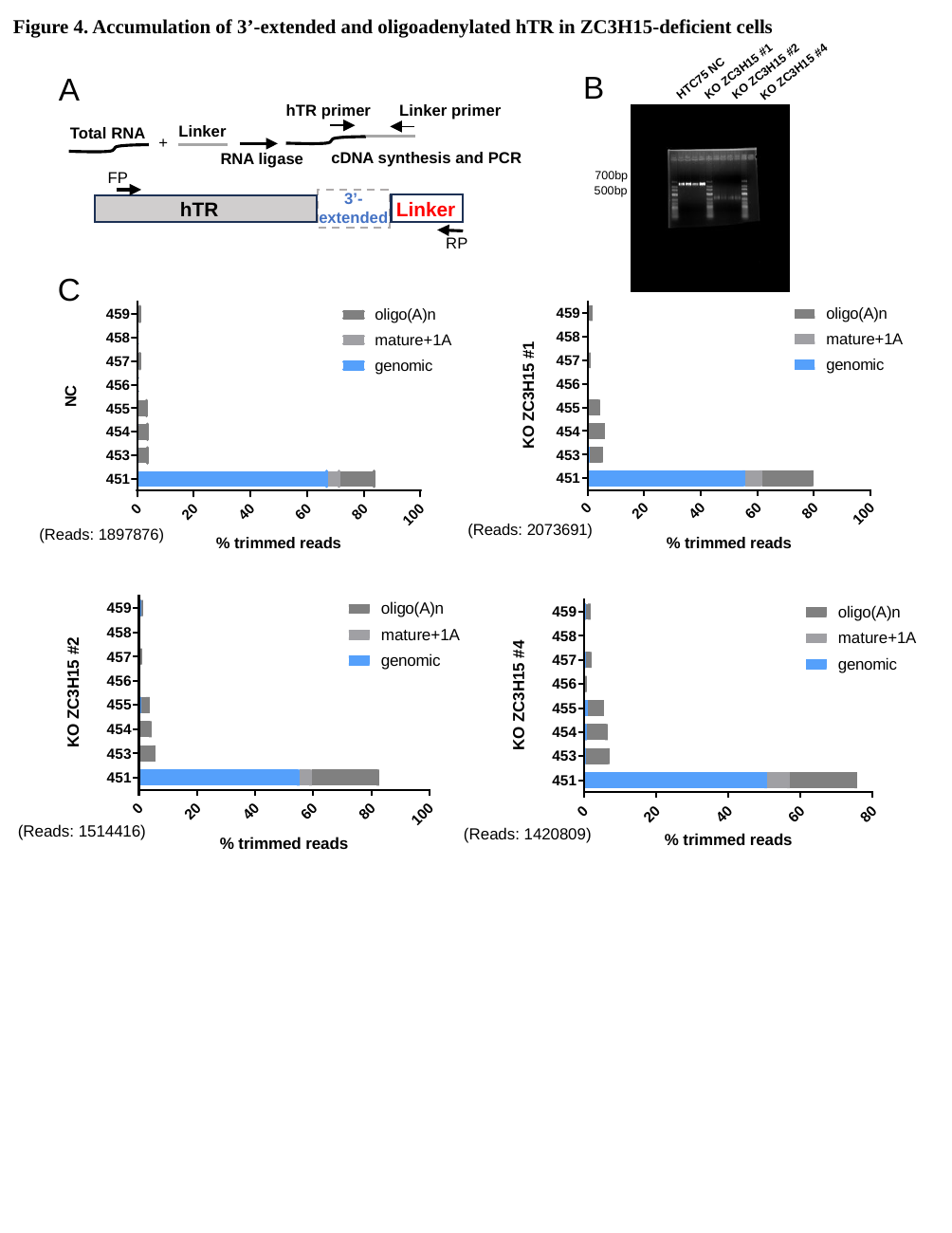

Figure 4. Accumulation of 3’-extended and oligoadenylated hTR in ZC3H15-deficient cells
KO ZC3H15 #2
KO ZC3H15 #1
KO ZC3H15 #4
HTC75 NC
B
700bp
500bp
A
hTR primer
Linker primer
Linker
Total RNA
+
cDNA synthesis and PCR
RNA ligase
FP
3’-
extended
hTR
Linker
RP
C

## Slide 5
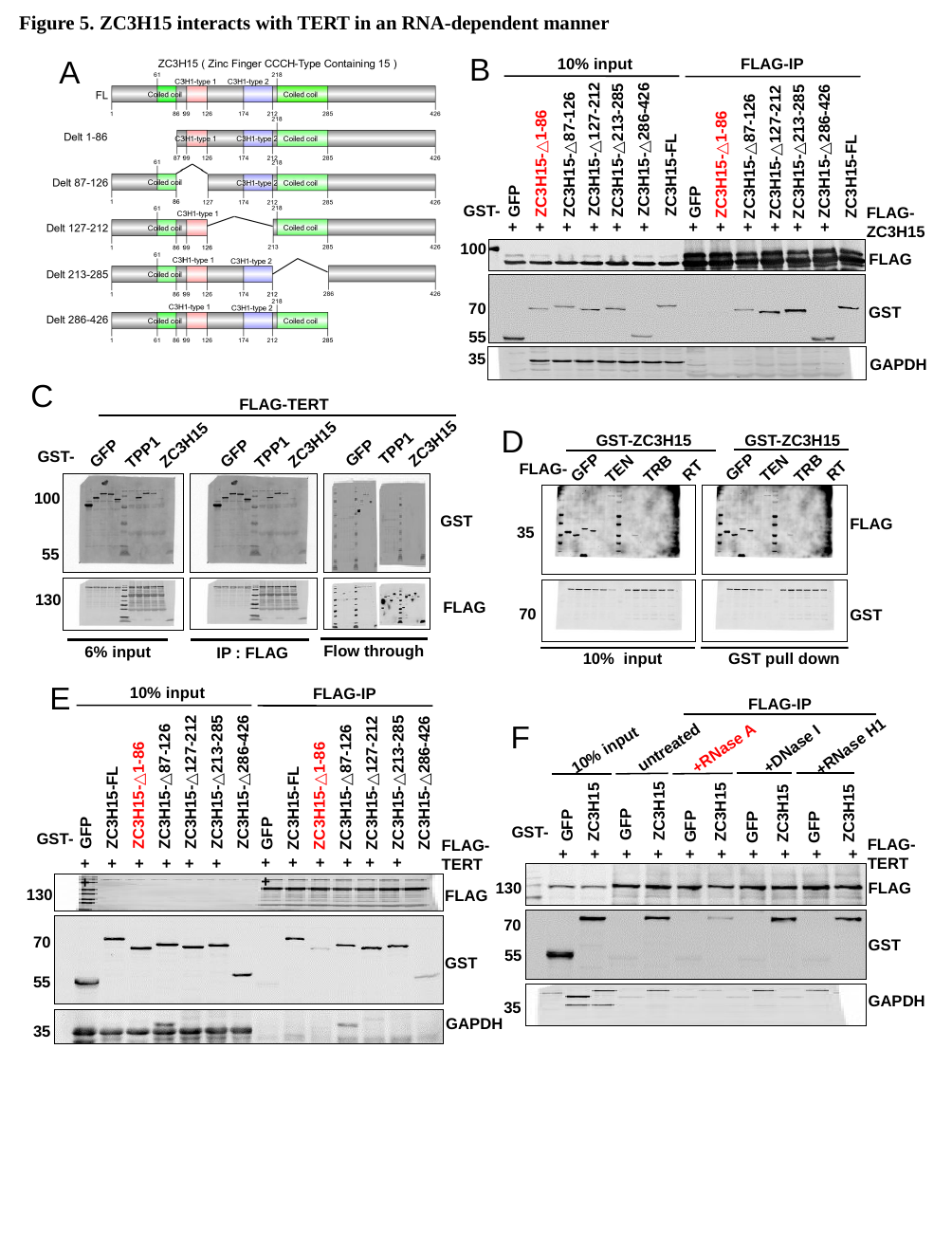

Figure 5. ZC3H15 interacts with TERT in an RNA-dependent manner
10% input
FLAG-IP
ZC3H15-△87-126
ZC3H15-△87-126
ZC3H15-△286-426
ZC3H15-△286-426
ZC3H15-△127-212
ZC3H15-△213-285
ZC3H15-△213-285
ZC3H15-△127-212
ZC3H15-△1-86
ZC3H15-△1-86
ZC3H15-FL
ZC3H15-FL
GFP
GFP
GST-
FLAG-ZC3H15
+ + + + + + +
+ + + + + + +
FLAG
GST
GAPDH
B
100
70
55
35
A
C
FLAG-TERT
ZC3H15
TPP1
GFP
ZC3H15
TPP1
GFP
D
GST-ZC3H15
GFP
TRB
TEN
RT
GST-ZC3H15
GFP
TRB
TEN
RT
ZC3H15
TPP1
GFP
GST-
FLAG-
100
GST
FLAG
35
55
130
FLAG
70
GST
Flow through
6% input
IP : FLAG
GST pull down
10% input
10% input
FLAG-IP
ZC3H15-△87-126
ZC3H15-△87-126
ZC3H15-△286-426
ZC3H15-△286-426
ZC3H15-△127-212
ZC3H15-△213-285
ZC3H15-△127-212
ZC3H15-△213-285
ZC3H15-△1-86
ZC3H15-△1-86
ZC3H15-FL
ZC3H15-FL
GFP
GFP
GST-
FLAG-TERT
+ + + + + + +
+ + + + + + +
FLAG
GST
GAPDH
E
130
70
55
35
FLAG-IP
+DNase I
untreated
+RNase A
+RNase H1
10% input
ZC3H15
ZC3H15
ZC3H15
ZC3H15
ZC3H15
GFP
GFP
GFP
GFP
GFP
GST-
FLAG-TERT
+ + + + + + + + + +
FLAG
GST
GAPDH
F
130
70
55
35

## Slide 6
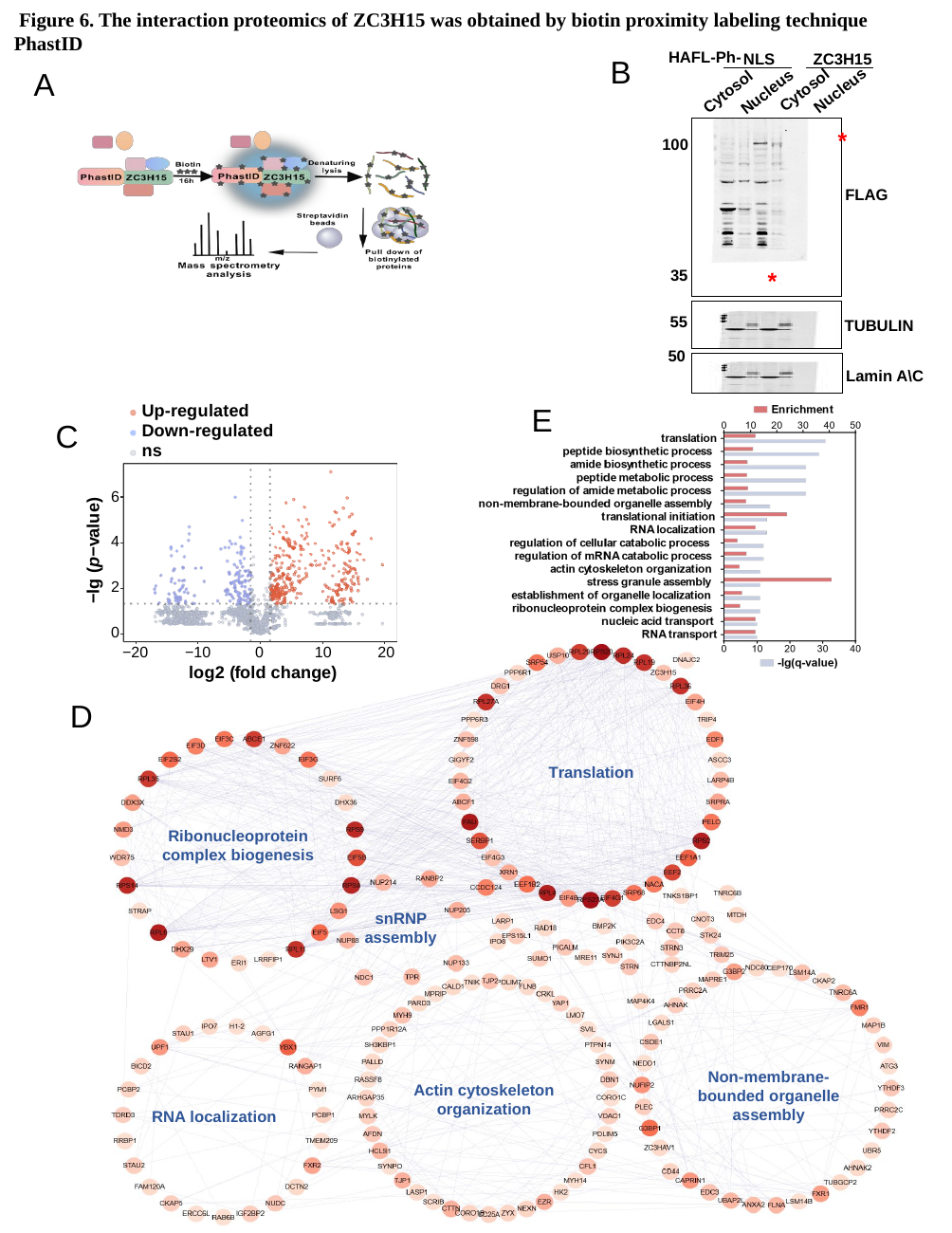

Figure 6. The interaction proteomics of ZC3H15 was obtained by biotin proximity labeling technique PhastID
HAFL-Ph-
NLS
ZC3H15
Nucleus
Cytosol
Cytosol
Nucleus
FLAG
TUBULIN
Lamin A\C
100
35
55
50
B
*
*
A
E
C
Translation
Ribonucleoprotein complex biogenesis
snRNP assembly
Non-membrane-bounded organelle assembly
Actin cytoskeleton organization
RNA localization
D

## Slide 7
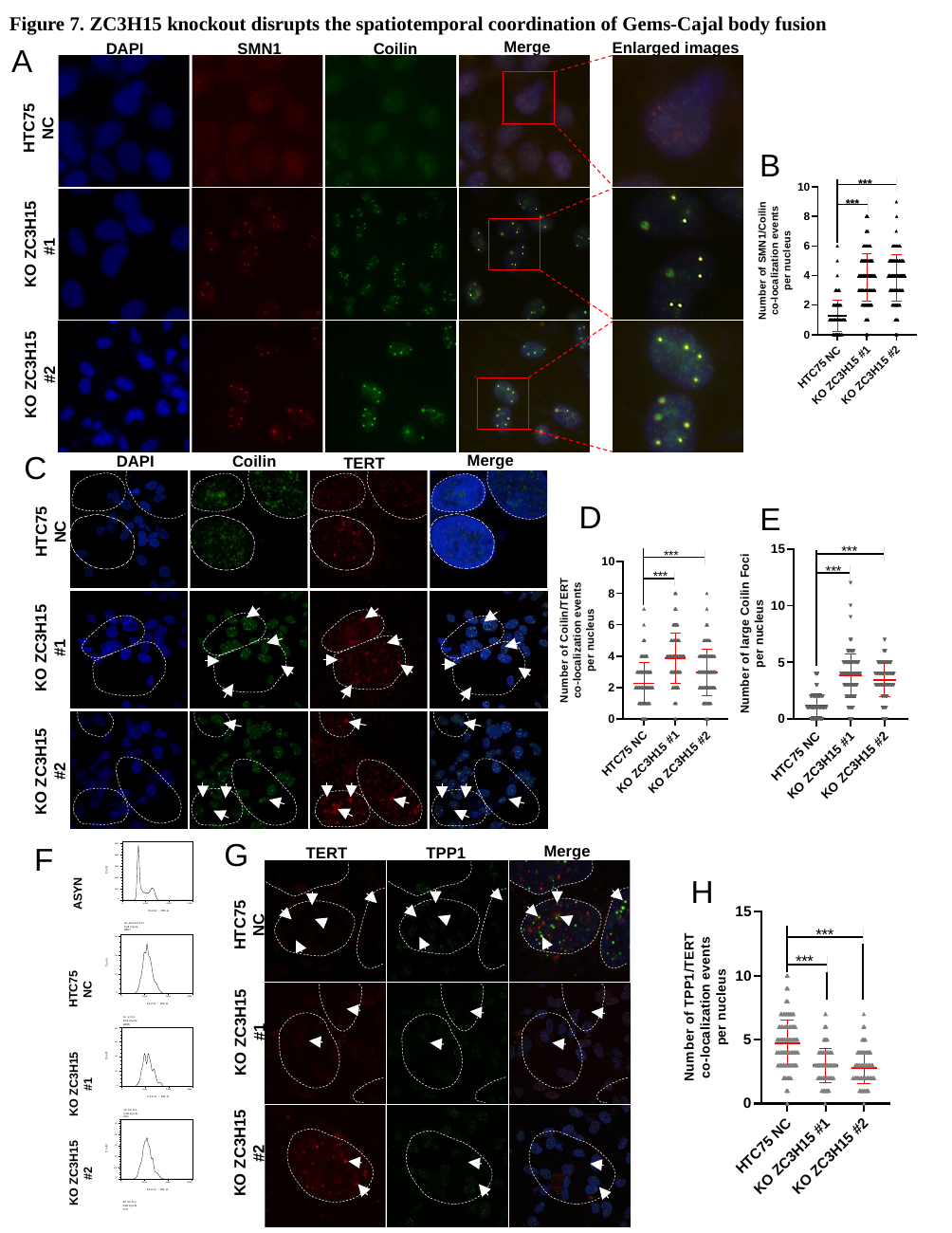

Figure 7. ZC3H15 knockout disrupts the spatiotemporal coordination of Gems-Cajal body fusion
Merge
DAPI
SMN1
Coilin
HTC75
NC
KO ZC3H15
#1
KO ZC3H15
#2
A
Enlarged images
B
C
Merge
DAPI
Coilin
TERT
HTC75
NC
KO ZC3H15
#1
KO ZC3H15
#2
D
E
G
Merge
TERT
TPP1
HTC75
NC
KO ZC3H15
#1
KO ZC3H15
#2
F
ASYN
HTC75
NC
KO ZC3H15
#1
KO ZC3H15
#2
H
